# Whole-heart electromechanical simulations using Latent Neural Ordinary Differential Equations

**DOI:** 10.1038/s41746-024-01084-x

**Published:** 2024-04-11

**Authors:** Matteo Salvador, Marina Strocchi, Francesco Regazzoni, Christoph M. Augustin, Luca Dede’, Steven A. Niederer, Alfio Quarteroni

**Affiliations:** 1 Institute for Computational and Mathematical Engineering, Stanford University, California, CA USA; 2 School of Biomedical Engineering and Imaging Sciences, King’s College London, London, UK; 3 MOX, Department of Mathematics, Politecnico di Milano, Milan, Italy; 4 National Heart and Lung Institute, Imperial College London, London, UK; 5 Institute of Biophysics, Medical University of Graz, Graz, Austria; 6 BioTechMed-Graz, Graz, Austria; 7 The Alan Turing Institute, London, UK; 8 École Polytechnique Fédérale de Lausanne, Lausanne, Switzerland

**Keywords:** Computational science, Computational models

## Abstract

Cardiac digital twins provide a physics and physiology informed framework to deliver personalized medicine. However, high-fidelity multi-scale cardiac models remain a barrier to adoption due to their extensive computational costs. Artificial Intelligence-based methods can make the creation of fast and accurate whole-heart digital twins feasible. We use Latent Neural Ordinary Differential Equations (LNODEs) to learn the pressure-volume dynamics of a heart failure patient. Our surrogate model is trained from 400 simulations while accounting for 43 parameters describing cell-to-organ cardiac electromechanics and cardiovascular hemodynamics. LNODEs provide a compact representation of the 3D-0D model in a latent space by means of an Artificial Neural Network that retains only 3 hidden layers with 13 neurons per layer and allows for numerical simulations of cardiac function on a single processor. We employ LNODEs to perform global sensitivity analysis and parameter estimation with uncertainty quantification in 3 hours of computations, still on a single processor.

## Introduction

Cardiac digital twins integrate physiological and pathological patient-specific data to monitor, analyze and forecast patient disease progression and outcomes. High-fidelity multi-scale and anatomically accurate models are available but require extensive high-performance computing resources to run, which limit their clinical translation^1^. Over the past years, these mathematical models evolved from an electromechanical description of the human ventricular activity in idealized shapes^2,3^ and realistic geometries^4–7^, while also addressing diseased conditions^8–12^, to whole-heart function^13–18^. Nevertheless, running many electromechanical simulations still entail high computational costs, hindering the development and application of cardiac digital twins. The use of Machine Learning tools, such as Gaussian Processes Emulators^19^ and Artificial Neural Networks (ANNs)^20,21^, allows to create efficient surrogate models that can be employed in many-query applications^22^, such as sensitivity analysis and parameter inference^23–25^. In the framework of digital twinning and personalized medicine, bridging the chasm between the need for a supercomputer^26–29^ and performing accurate numerical simulations on a standard computer^30–33^ would have a tremendous impact on the clinical adoption of computational cardiology.

In this work, we develop a Scientific Machine Learning method to build a comprehensive surrogate model involving both cardiac and cardiovascular function. Specifically, we train a system of Latent Neural Ordinary Differential Equations (LNODEs)^20,34,35^ that learns the pressure-volume transients of a heart failure patient while varying 43 model parameters that describe cardiac electrophysiology, active and passive mechanics, and cardiovascular fluid dynamics, by employing 400 3D-0D closed-loop electromechanical training simulations. We design a suitable loss function that is minimized during the tuning process of the ANN parameters, which entails small relative errors of LNODEs, i.e., from 2% to 6%, when the number of training samples is small compared to the dimensionality of the parameter space and the explored model variability. These LNODEs enable four-chamber heart numerical simulations on a standard computer by encoding pressure-volume dynamics while spanning electro-mechano-fluid model parameters throughout the cardiovascular system. Furthermore, they can be easily trained on a single central processing unit (CPU).

We use the trained LNODEs to perform global sensitivity analysis (GSA) and robust parameter estimation with uncertainty quantification (UQ)^23,24^. For the former, we observe how model parameters impact the variability of scalar quantities of interest (QoIs) retrieved from the pressure-volume time traces, by considering both first-order and high-order interactions via Sobol indices^36^. For the latter, we combine two Bayesian statistics methods, i.e., Maximum a Posteriori (MAP) estimation and Hamiltonian Monte Carlo (HMC)^34,37,38^, where we exploit efficient matrix-free adjoint-based methods, automatic differentiation and vectorization^34^. In particular, we design several test cases where we calibrate tens of model parameters by matching the pressure and volume time traces, that are time-dependent QoIs, coming from 5 unseen 3D-0D numerical simulations for the trained ANN. GSA and parameter estimation with UQ can be carried out in 3 hours of computations by using a single core standard laptop.

## Results

**Fig. 1:**
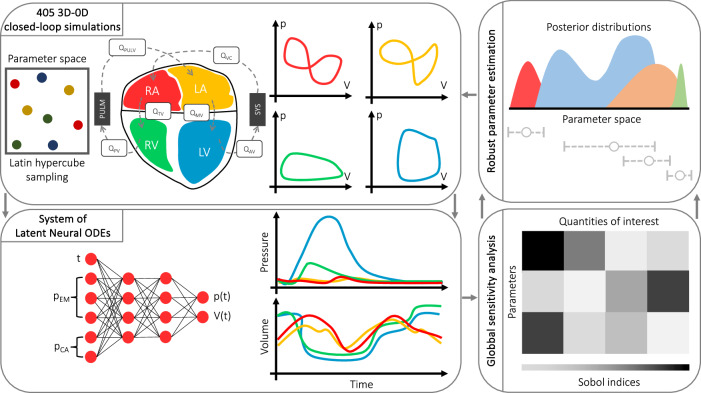
Sketch of the computational pipeline. We perform several 3D-0D closed-loop four-chamber heart electromechanical simulations. We build an accurate and efficient ANN-based surrogate model of the whole cardiovascular function by means of LNODEs. We carry out GSA to understand how each model parameter influences different QoIs extracted from the simulated pressure-volume loops. We robustly estimate many model parameters from time-dependent QoIs. Fully personalized 3D-0D numerical simulations can be performed after parameter calibration with UQ.

We display the whole computational pipeline in Fig. 1.

- Top-left: we use a database of *N*_sims_ = 405 electromechanical simulations generated by a personalized anatomy four-chamber heart model from a heart failure patient (see Supplementary Material 1), where we vary ${N}_{{{{\mathcal{P}}}}}=43$ parameters that describe cell, tissue, whole-heart and cardiovascular system material properties and boundary conditions. For all the numerical simulations, we run 5 heartbeats in sinus rhythm and we perform our analysis on the pressure and volume transients of the last cardiac cycle. We refer to Supplementary Material 2 for all the details about the four-chamber physics-based mathematical model and the numerical settings of these simulations. All the information regarding model parameters can be found in Supplementary Material 3.
- Bottom-left: we employ *N*_train, valid_ = 400 simulations to tune the LNODEs hyperparameters. This surrogate model learns the atrial and ventricular pressure-volume temporal dynamics of the last cardiac cycle only, while receiving time and model parameters as inputs. We perform *K*-fold cross validation with *K* = 10 for the training-validation splitting. We detail the whole optimization process to get the final values of the LNODEs hyperparameters in Supplementary Material 4. We evaluate the accuracy of the trained LNODEs on a testing dataset consisting of the remaining *N*_test_ = 5 numerical simulations.
- Bottom-right: we employ the trained LNODEs to perform GSA.
- Top-right: we estimate model parameters with UQ on *N*_test_ = 5 numerical simulations by means of the trained LNODEs.

### Learning atrial and ventricular pressure-volume loops

Automatic hyperparameters tuning with *K*-fold cross validation leads to an optimal ANN architecture comprising 3 hidden layers and 13 neurons per hidden layer. The optimal number of states is set to *N*_*z*_ = 8, i.e., no latent variables are selected. This is motivated by the trade-off between the size of the training set *N*_train, valid_ with respect to the number of parameters ${N}_{{{{\mathcal{P}}}}}$, i.e., a thrifty system of LNODEs with no additional hidden variables **z**_latent_(*t*) is selected to avoid overfitting. More details regarding LNODEs training and hyperparameters tuning are given in Supplementary Material 4.

**Fig. 2:**
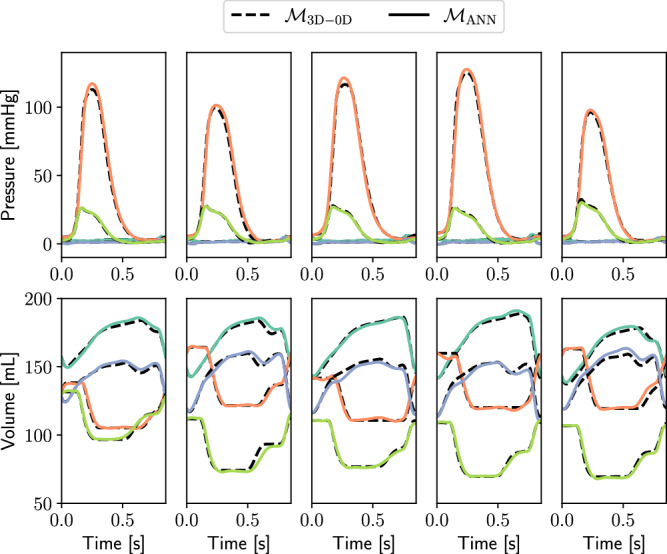
Pressure and volume time transients obtained with model ${{{{\mathcal{M}}}}}_{3{{{\rm{D}}}}{{\mbox{-}}}0{{{\rm{D}}}}}$ (dashed lines), compared to those obtained with model ${{{{\mathcal{M}}}}}_{{{{\rm{ANN}}}}}$ (solid lines), on the testing samples (*N*_test_ = 5). Light blue: LA, orange: LV, blue: RA, green: RV.

In Table 1, we report the Normalized Root Mean Square Error (NRMSE) and *R*^2^ coefficients associated with the LA, LV, RA, and RV pressure-volume time traces provided by LNODEs. These values are obtained by considering a test set comprised of *N*_test_ = 5 electromechanical simulations. The accuracy obtained by our surrogate model in reproducing the cardiac outputs is high, manifesting testing errors that approximately range from 2% to 6% for all time-dependent QoIs. The good match between models ${{{{\mathcal{M}}}}}_{3{{{\rm{D}}}}{{\mbox{-}}}0{{{\rm{D}}}}}$ and ${{{{\mathcal{M}}}}}_{{{{\rm{ANN}}}}}$ is also confirmed by Fig. 2, where atrial and ventricular pressure-volume traces present a good overlap on the whole testing set.

**Table 1:** Testing errors and *R*^2^ coefficients on the time-dependent outputs of the trained LNODEs system

		Pressure			
		*p*_LA_(*t*)	*p*_LV_(*t*)	*p*_RA_(*t*)	*p*_RV_(*t*)
${{{{\mathcal{M}}}}}_{3{{{\rm{D}}}}{{\mbox{-}}}0{{{\rm{D}}}}}$ vs ${{{{\mathcal{M}}}}}_{{{{\rm{ANN}}}}}$	NRMSE	0.028	0.022	0.022	0.021
	R^2^	99.23	99.82	98.85	99.81

		Volume			
		*V*_LA_(*t*)	*V*_LV_(*t*)	*V*_RA_(*t*)	*V*_RV_(*t*)
${{{{\mathcal{M}}}}}_{3{{{\rm{D}}}}{{\mbox{-}}}0{{{\rm{D}}}}}$ vs ${{{{\mathcal{M}}}}}_{{{{\rm{ANN}}}}}$	NRMSE	0.036	0.030	0.054	0.026
	R^2^	99.36	99.50	97.97	99.58

### Global sensitivity analysis

**Fig. 3:**
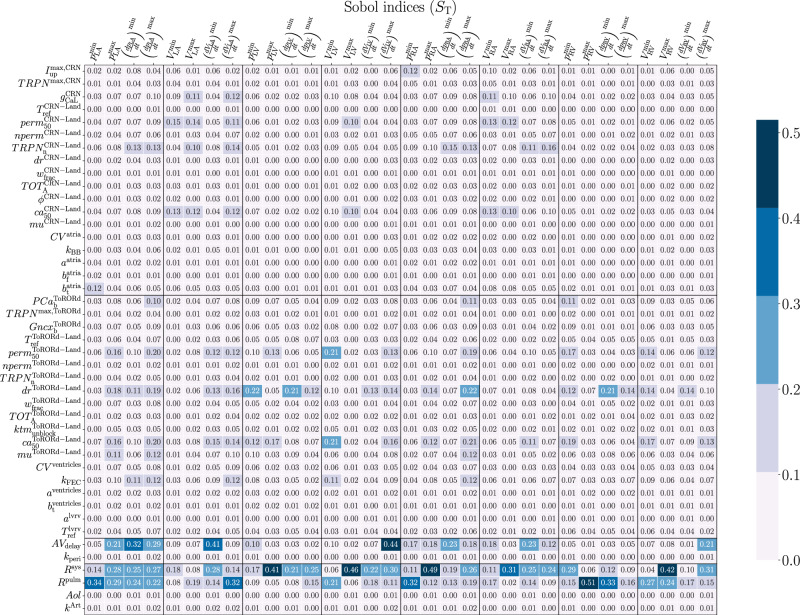
Total-effect Sobol indices computed by exploiting model ${{{{\mathcal{M}}}}}_{{{{\rm{ANN}}}}}$. For a detailed definition of all model parameters and QoIs, we refer to Supplementary Material 3.

Figure 3 shows the total-effect Sobol indices. We consider a parameter to be relevant if the associated Sobol indices are greater than 10^−1^ for at least one QoI. We notice that, as expected from a physiological point of view, some model parameters are compartmentalized, i.e., cell-to-organ level values coming from a certain compartment of the cardiocirculatory system mostly explain the variability of QoIs that are specific to that region. Indeed, some parameters of the CRN-Land model, such as atrial calcium/troponin complex when 50% of crossbridges are blocked $per{m}_{{{{\rm{50}}}}}^{{{{\rm{CRN-Land}}}}}$, atrial *Ca*^2+^-troponin cooperativity $TRP{N}_{{{{\rm{n}}}}}^{{{{\rm{CRN-Land}}}}}$ and atrial reference *Ca*^2+^ sensitivity $c{a}_{{{{\rm{50}}}}}^{{{{\rm{CRN-Land}}}}}$, or of the Guccione model, such as atrial stiffness in the transverse plane ${b}_{{{{\rm{t}}}}}^{{{{\rm{atria}}}}}$, have an important role in determining atrial behavior. Similar considerations occur for the ventricular part of the heart, where the most important parameters are related to the ToRORd-Land model. Nevertheless, it is important to notice the interplay between some ventricular parameters of the ToRORd-Land model at the cellular scale, such as ventricular steady-state duty ratio *dr*^ToRORd-Land^, ventricular calcium/troponin complex when 50% of crossbridges are blocked $per{m}_{{{{\rm{50}}}}}^{{{{\rm{ToRORd-Land}}}}}$ and ventricular reference *Ca*^2+^ sensitivity $c{a}_{{{{\rm{50}}}}}^{{{{\rm{ToRORd-Land}}}}}$ and the atrial function. This is a particularly interesting insight into cardiac physiology that can be clearly unraveled using this type of comprehensive sensitivity analysis. We highlight that, as expected, some model parameters, such as atrioventricular delay *AV*_delay_, systemic resistance *R*^sys^ and pulmonary resistance *R*^pulm^ strongly affect all QoIs, whereas others, such as the pericardial coefficient *k*_peri_, as well as aorta parameters (length *Aol*, stiffness *k*^Art^), have a minor role in determining all QoIs.

Finally, we remark that Sobol indices are affected by the amplitude of the ranges in which the parameters are varied. In particular, the wider the range associated with a parameter, the greater the associated Sobol indices will be, as the parameter in question potentially generates greater variability in the QoI. Therefore, we stress that our results are valid for the specific ranges we used.

### Robust parameter estimation

In the context of parameter calibration, a preliminary GSA allows to determine the identifiability of model parameters according to the provided QoIs. Based on the results obtained from global sensitivity analysis, we design 4 in silico test cases to show the robustness and flexibility of our parameter calibration process, which is driven by a combined use of MAP estimation and HMC starting from time-dependent QoIs. In Table 2, we report the observed pressure-volume time traces and estimated model parameters for each test case. In ${{{{\mathcal{T}}}}}_{{{{\rm{LV}}}}}$ and ${{{{\mathcal{T}}}}}_{{{{\rm{ventricles}}}}}$, we estimate model parameters related to the ventricular and cardiovascular function starting from time-dependent QoIs localized in the ventricles. In ${{{{\mathcal{T}}}}}_{{{{\rm{atria}}}}}$, we calibrate model parameters over the whole cardiac function and cardiocirculatory network by only considering atrial observations. Finally, we challenge our surrogate model by taking all cardiac pressures and volumes over time and by estimating 11 model parameters.

**Table 2:** Summary of the 4 in silico test cases for parameter calibration

Test case	Time-dependent QoIs	Estimated model parameters
${{{{\mathcal{T}}}}}_{{{{\rm{LV}}}}}$	*V*_LV_	*dr*^ToRORd-Land^, $c{a}_{{{{\rm{50}}}}}^{{{{\rm{ToRORd-Land}}}}}$, *R*^sys^, *R*^pulm^
${{{{\mathcal{T}}}}}_{{{{\rm{ventricles}}}}}$	*V*_LV_, *V*_RV_	*dr*^ToRORd-Land^, $c{a}_{{{{\rm{50}}}}}^{{{{\rm{ToRORd-Land}}}}}$, $per{m}_{{{{\rm{50}}}}}^{{{{\rm{ToRORd-Land}}}}}$, *R*^sys^, *R*^pulm^
${{{{\mathcal{T}}}}}_{{{{\rm{atria}}}}}$	*V*_LA_, *V*_RA_	*dr*^ToRORd-Land^, $per{m}_{{{{\rm{50}}}}}^{{{{\rm{ToRORd-Land}}}}}$, $c{a}_{{{{\rm{50}}}}}^{{{{\rm{CRN-Land}}}}}$, $TRP{N}_{{{{\rm{n}}}}}^{{{{\rm{CRN-Land}}}}}$, ${g}_{{{{\rm{CaL}}}}}^{{{{\rm{CRN}}}}}$, ${b}_{{{{\rm{t}}}}}^{{{{\rm{atria}}}}}$, *R*^sys^, *R*^pulm^
${{{{\mathcal{T}}}}}_{{{{\rm{all}}}}}$	*p*_LA_, *p*_RA_, *p*_LV_, *p*_RV_, *V*_LA_, *V*_RA_, *V*_LV_, *V*_RV_	*dr*^ToRORd-Land^, $per{m}_{{{{\rm{50}}}}}^{{{{\rm{ToRORd-Land}}}}}$, $c{a}_{{{{\rm{50}}}}}^{{{{\rm{ToRORd-Land}}}}}$, $c{a}_{{{{\rm{50}}}}}^{{{{\rm{CRN-Land}}}}}$, *CV*^ventricles^, $TRP{N}_{{{{\rm{n}}}}}^{{{{\rm{CRN-Land}}}}}$, *k*_FEC_, ${g}_{{{{\rm{CaL}}}}}^{{{{\rm{CRN}}}}}$, ${b}_{{{{\rm{t}}}}}^{{{{\rm{atria}}}}}$, *R*^sys^, *R*^pulm^

${g}_{{{{\rm{CaL}}}}}^{{{{\rm{CRN}}}}}$: AT conductance of L-type *Ca*^2+^ current, $TRP{N}_{{{{\rm{n}}}}}^{{{{\rm{CRN-Land}}}}}$: AT *Ca*^2+^-troponin cooperativity, $c{a}_{{{{\rm{50}}}}}^{{{{\rm{CRN-Land}}}}}$: AT reference *Ca*^2+^ sensitivity, ${b}_{{{{\rm{t}}}}}^{{{{\rm{atria}}}}}$: AT stiffness in the transverse plane, $per{m}_{{{{\rm{50}}}}}^{{{{\rm{ToRORd-Land}}}}}$: VE calcium/troponin complex when 50% of crossbridges are blocked, *dr*^ToRORd-Land^: VE steady-state duty ratio, $c{a}_{{{{\rm{50}}}}}^{{{{\rm{ToRORd-Land}}}}}$: VE reference *Ca*^2+^ sensitivity, *CV*^ventricles^: VE conduction velocity in the fiber direction, *k*_FEC_: fast endocardial layer scaling factor, *R*^sys^: systemic resistance scaling factor, *R*^pulm^: pulmonary resistance scaling factor, AT: atrial, VE: ventricular.

**Fig. 4:**
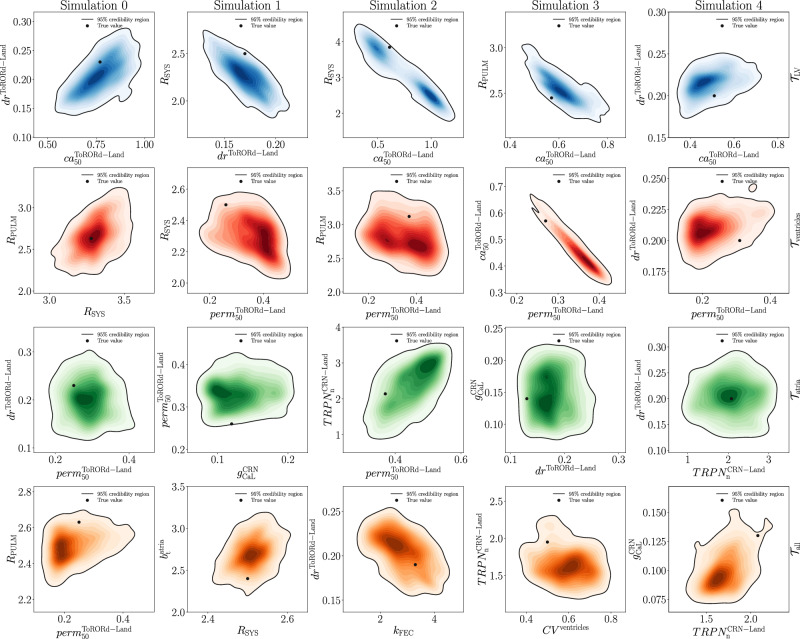
Two-dimensional views of the posterior distribution estimated by means of HMC for each test case (rows) and *N*_test_ = 5 electromechanical simulations (columns). Different colors are associated to ${{{{\mathcal{T}}}}}_{{{{\rm{LV}}}}}$, ${{{{\mathcal{T}}}}}_{{{{\rm{ventricles}}}}}$, ${{{{\mathcal{T}}}}}_{{{{\rm{atria}}}}}$, ${{{{\mathcal{T}}}}}_{{{{\rm{all}}}}}$.

We perform parameter estimation with UQ on *N*_test_ = 5 electromechanical simulations that are unseen by the trained LNODEs. Figure 4 shows some two-dimensional views of the posterior distribution for each test case and for all *N*_test_ numerical simulations. We notice that the true parameter values are contained inside the 95% credibility regions. Moreover, by using Bayesian statistics we are able to capture relationships among model parameters. In particular, in Fig. 4 we consider different pairs of model parameters for each test case and numerical simulation to maximize the number of interactions. For instance, pulmonary resistance scaling factor *R*^pulm^ and ventricular steady-state duty ratio *dr*^ToRORd-Land^ are positively correlated with systemic resistance scaling factor *R*^sys^ and ventricular reference *Ca*^2+^ sensitivity $c{a}_{{{{\rm{50}}}}}^{{{{\rm{ToRORd-Land}}}}}$, respectively, while ventricular steady-state duty ratio *dr*^ToRORd-Land^ and ventricular reference *Ca*^2+^ sensitivity $c{a}_{{{{\rm{50}}}}}^{{{{\rm{ToRORd-Land}}}}}$ are negatively correlated with fast endocardial layer scaling factor *k*_FEC_ and ventricular calcium/troponin complex when 50% of crossbridges are blocked $per{m}_{{{{\rm{50}}}}}^{{{{\rm{ToRORd-Land}}}}}$, respectively. We notice that, in some cases, cell-based atrial and ventricular parameters may be correlated, as it happens for atrial *Ca*^2+^-troponin cooperativity $TRP{N}_{{{{\rm{n}}}}}^{{{{\rm{CRN-Land}}}}}$ and ventricular calcium/troponin complex when 50% of crossbridges are blocked $per{m}_{{{{\rm{50}}}}}^{{{{\rm{ToRORd-Land}}}}}$, while in most situations, such as with ventricular steady-state duty ratio *dr*^ToRORd-Land^ and atrial *Ca*^2+^-troponin cooperativity $TRP{N}_{{{{\rm{n}}}}}^{{{{\rm{CRN-Land}}}}}$, there is no interaction. We also remark that this kind of relationships may be unraveled among different physical problems. For instance, this occurs between cardiovascular hemodynamics (systemic resistance scaling factor *R*^sys^) and the ventricular cell tension model (ventricular steady-state duty ratio *dr*^ToRORd-Land^). The aforementioned interactions among model parameters can be quite interesting and surprising from a physiological perspective, especially when they involve very different cardiovascular compartments of this complex multiscale and multiphysics mathematical model. For the sake of completeness, in Table 3, we report the identified parameter values of ventricular steady-state duty ratio *dr*^ToRORd-Land^, systemic resistance scaling factor *R*^sys^ and pulmonary resistance scaling factor *R*^pulm^ for all test cases, with respect to the first testing simulation. We see that the true values of the parameters are always contained inside the interval defined by median plus/minus interquartile range. We refer to Supplementary Material 6 for the tables containing similar results and comparisons for all test cases (${{{{\mathcal{T}}}}}_{{{{\rm{LV}}}}}$, ${{{{\mathcal{T}}}}}_{{{{\rm{ventricles}}}}}$, ${{{{\mathcal{T}}}}}_{{{{\rm{atria}}}}}$ and ${{{{\mathcal{T}}}}}_{{{{\rm{all}}}}}$) with all the relevant model parameters over the *N*_test_ electromechanical simulations.

**Table 3:** True value and median with interquartile range (between brackets) associated to the estimated values of ventricular steady-state duty ratio *dr*^ToRORd-Land^, systemic resistance scaling factor *R*^sys^ and pulmonary resistance scaling factor *R*^pulm^ during HMC for the first numerical simulation of the testing set

Parameter	Ground truth	${{{{\mathcal{T}}}}}_{{{{\rm{LV}}}}}$	${{{{\mathcal{T}}}}}_{{{{\rm{ventricles}}}}}$	${{{{\mathcal{T}}}}}_{{{{\rm{atria}}}}}$	${{{{\mathcal{T}}}}}_{{{{\rm{all}}}}}$
*dr*^ToRORd-Land^	0.23	0.21 (0.05)	0.23 (0.03)	0.20 (0.05)	0.27 (0.04)
*R*^sys^	3.28	3.28 (0.49)	3.30 (0.13)	3.32 (0.27)	3.18 (0.11)
*R*^pulm^	2.63	2.70 (0.43)	2.66 (0.26)	2.99 (0.49)	2.51 (0.15)

## Discussion

In this work, we propose a surrogate model based on LNODEs to learn the pressure-volume temporal dynamics of 3D-0D closed-loop four-chamber heart electromechanical simulations^23^. The geometry is retrieved from a heart failure patient and some functional aspects have been incorporated in the electromechanical simulations. Specifically, we get QRS duration from 12-lead electrocardiograms, ventricle reference tension for active contraction and heart rate, in order to achieve pressure-volume loops that are consistent with measured peak pressure and pressure transient duration^25^. In particular, starting from 400 numerical simulations, we create an anatomy-specific surrogate model by leveraging LNODEs. These are defined by a lightweight feedforward fully-connected ANN containing 3 hidden layers and 13 neurons per layer. LNODEs retain the variability of 43 model parameters that describe electrophysiology, active and passive mechanics, and hemodynamics, both at the cell level and organ scale, and covering a wide range of pressure and volume values (see Figures in Supplementary Material 2). Indeed, LNODEs allows to capture complex dynamics with a small number of tunable parameters. This paradigm, opposed to large machine learning models that work in the overparameterization regime, has proven to be very effective and robust, showing great generalization properties. Some other examples are given by Latent Dynamics Networks^39^ or Liquid Neural Networks^40^, which are built on top of LNODEs to account for complex space-time processes exhibiting abrupt changes by using very simple architectures and small latent spaces.

The generation of such a comprehensive training dataset poses an incredible technological challenge itself in the scientific community^25^, and in recent years different surrogate models of cardiac electromechanics based on emulators have been proposed in the literature to provide fast and accurate evaluations based on computationally expensive physics-based mathematical models^19,25,41–43^. These emulators are built on a collection of pre-computed numerical simulations obtained by sampling the parameter space, similarly to what has been done in this work. However, they only fit a static map between model parameters and pointwise QoIs extracted from the numerical simulations. On the other hand, LNODEs present a higher representational power, because they encode time dependent electromechanical simulations instead of pointwise QoIs, while also requiring a smaller amount of data to reach a prescribed accuracy^23^. This is why this paper provides a comprehensive surrogate model embracing cardiac and cardiovascular function.

The choice of 400 numerical simulations for the training of LNODEs allows to get consistently low validation and testing errors, in the order of 2% to 6%, even in areas of the parameter space that are sparsely covered by the training samples (see Figure in Supplementary Material 3). The error remains within these bounds even if we increase the dimension of the testing set while decreasing the one of the training set (see Supplementary Material 9). In addition, LNODEs provide better generalization properties compared to Gaussian processes emulators, especially for ventricular function (see Supplementary Material 8).

LNODEs require a small amount of computational resources and enable several applications of interest in a very fast and accurate manner. Indeed, as reported in Table 4, running the training phase of the ANN along with GSA and robust parameter estimation on a single core standard laptop just requires 13 h of computations. We remark that this time can be reduced with a multi-core implementation. On the other hand, employing the 3D-0D model ${{{{\mathcal{M}}}}}_{3{{{\rm{D}}}}{{\mbox{-}}}0{{{\rm{D}}}}}$ for the same computational pipeline would entail very significant costs. The overall speed-up with the surrogate model ${{{{\mathcal{M}}}}}_{{{{\rm{ANN}}}}}$ is equal to 1718x. Furthermore, after the parameter calibration process, a whole-heart electromechanical simulation can run via high-performance computing with the estimated model parameters, showing all relevant space-time fields, such as transmembrane potential, active contraction, displacement and stresses. This would provide relevant insights across the whole high-fidelity cardiac model.

**Table 4:** Summary of the approximated computational times to perform GSA and parameter estimation with UQ. 3D-0D closed-loop model ${{{{\mathcal{M}}}}}_{3{{{\rm{D}}}}{{\mbox{-}}}0{{{\rm{D}}}}}$ (top) and LNODEs ${{{{\mathcal{M}}}}}_{{{{\rm{ANN}}}}}$ (bottom)

Task	Computational resources	Execution time
$${{{{\mathcal{M}}}}}_{3{{{\rm{D}}}}{{\mbox{-}}}0{{{\rm{D}}}}}$$		
Single simulation (5 heartbeats)	512 cores	6 h and 20 min
GSA (704’000 simulations)	512 cores	508 years
Parameter estimation with UQ (750 heartbeats)	512 cores	0.5 years
		Total: 508.5 years
$${{{{\mathcal{M}}}}}_{{{{\rm{ANN}}}}}$$		
Training dataset generation (405 simulations)	512 cores	106 days and 21 h
Reduced-order model training	1 core	10 h
GSA (704’000 heartbeats)	1 core	2 h
Parameter estimation with UQ (750 heartbeats)	1 core	1 h
		Total: 108 days

It is interesting to note that several model parameters related to electrophysiology, mechanics, and hemodynamics at the cell-to-organ scale have a significant impact on the pressure-volume loops. These model parameters can be inferred from the pressure-volume relationships using Bayesian parameter estimation with UQ, i.e., their true values are contained within the 95% credibility regions of the posterior distribution. In addition, bayesian statistics provides important insights by capturing cross-correlations between model parameters. This occurs even between different cardiovascular compartments, such as the systemic circulation and ventricular electromechanics. Our approach could be applied in a clinical setting by using clinically measured rather than computational pressure-volume loops to infer protein and cell-to-organ function directly from clinical data.

Even though this paper focuses on a single anatomy, it represents an important milestone towards the construction of emulators incorporating geometric variability. Indeed, the presented approach can be extended to cover patient variability, by incorporating statistical shape modeling^44,45^ or other ANN-based methods, such as Universal Solution Manifold Networks^46^ or generative deep learning techniques based on Signed Distance Fields^47,48^, within LNODEs, to encode different geometrical parameterizations. Furthermore, multiple pathological conditions and diagnoses can be taken into account by providing specific one-hot vectors as additional inputs to the ANN^47^. In this manner, we would run the numerical simulations with the biophysically detailed and anatomically accurate mathematical model just once and we would train an ANN that generalizes on multiple patients, while effectively capturing multi-physics and multi-scale knowledge. Moreover, even though patient-specific pressure-volume loops have not been considered in this work, we aim at adding this information as part of our computational pipeline for parameter calibration with UQ. The proposed method paves the way to extensions incorporating different anatomies and pathological conditions, which would potentially allow for a universal whole-heart simulator that might be readily deployed in clinical practice for fast and reliable personalized parameter calibration based on patient-specific data.

## Methods

### Ethics statement

The clinical data used in this study were collected as part of a clinical trial (REC reference: 14/WM/1069) approved by the West Midlands-Coventry & Warwickshire Research Ethics Committee.

### Learning atrial and ventricular pressure-volume loops

Following the model learning approach introduced in ref. ^20^, we build a system of LNODEs, i.e., a set of ordinary differential equations whose right hand side is represented by a feedforward fully-connected ANN, that learns the pressure-volume temporal dynamics of the 3D-0D closed-loop electromechanical model ${{{{\mathcal{M}}}}}_{3{{{\rm{D}}}}{{\mbox{-}}}0{{{\rm{D}}}}}$ in a latent space. All details regarding cardiac anatomy and the model ${{{{\mathcal{M}}}}}_{3{{{\rm{D}}}}{{\mbox{-}}}0{{{\rm{D}}}}}$, as well as the coupling to pressure-volume loops, are reported in Supplementary Material 1 and 2, respectively. In this framework, the four-chamber heart surrogate model ${{{{\mathcal{M}}}}}_{{{{\rm{ANN}}}}}$ reads:

1 $$\left\{\begin{array}{ll}\dfrac{d{{{\bf{z}}}}(t)}{dt}={{{\mathcal{ANN}}}}\left({{{\bf{z}}}}(t),\cos \left(\dfrac{2\pi (t-A{V}_{{{{\rm{delay}}}}})}{{T}_{{{{\rm{HB}}}}}}\right),\sin \left(\dfrac{2\pi (t-A{V}_{{{{\rm{delay}}}}})}{{T}_{{{{\rm{HB}}}}}}\right),{{{\boldsymbol{\theta }}}};{{{\bf{w}}}}\right)&\,{{\mbox{for}}}\,\,t\in (0,{T}_{{{{\rm{HB}}}}}],\\ {{{\bf{z}}}}(0)={{{{\bf{z}}}}}_{0},\end{array}\right.$$

where **z**_0_ is the vector of initial conditions. The ANN, with weights and biases encoded in ${{{\bf{w}}}}\in {{\mathbb{R}}}^{{N}_{w}}$, is defined by ${{{\mathcal{ANN}}}}:{{\mathbb{R}}}^{{N}_{z}+2+{N}_{{{{\mathcal{P}}}}}}\to {{\mathbb{R}}}^{{N}_{z}}$. Vector ${{{\boldsymbol{\theta }}}}\in {{{\boldsymbol{\Theta }}}}\subset {{\mathbb{R}}}^{{N}_{{{{\mathcal{P}}}}}}$ defines the model ${{{{\mathcal{M}}}}}_{3{{{\rm{D}}}}{{\mbox{-}}}0{{{\rm{D}}}}}$ parameters. Some examples of ***θ*** could be conductances of different ionic channels, myocardial conductivity, atrial and ventricular active tension or passive stiffness, and resistances of the systemic and pulmonary circulation. The reduced state vector ${{{\bf{z}}}}(t)\in {{\mathbb{R}}}^{{N}_{z}}$ contains the time-dependent pressure and volume variables of the left atrium (LA), right atrium (RA), left ventricle (LV) and right ventricle (RV), as well as additional latent variables without a direct physical interpretation, that is ${{{\bf{z}}}}(t)=[{{{{\bf{z}}}}}_{{{{\rm{physical}}}}}(t),{{{{\bf{z}}}}}_{{{{\rm{latent}}}}}(t)]={[{p}_{{{{\rm{LA}}}}}(t),{p}_{{{{\rm{LV}}}}}(t),{p}_{{{{\rm{RA}}}}}(t),{p}_{{{{\rm{RV}}}}}(t),{V}_{{{{\rm{LA}}}}}(t),{V}_{{{{\rm{LV}}}}}(t),{V}_{{{{\rm{RA}}}}}(t),{V}_{{{{\rm{RV}}}}}(t),{{{{\bf{z}}}}}_{{{{\rm{latent}}}}}(t)]}^{T}$. The ANN receives *N*_*z*_ state variables, ${N}_{{{{\mathcal{P}}}}}$ scalar parameters, and two periodic inputs. Indeed, even though LNODEs are just trained on the last cardiac cycle, the cosine and sine terms account for the heartbeat period *T*_HB_ and the atrioventricular delay *AV*_delay_ of whole-heart electromechanical simulations (see Supplementary Material 2 for further details). On the other hand, the vector of physics-based model parameters ***θ***, which involves cardiac electromechanics and cardiovascular hemodynamics, is not related to the time variable, as is the case for *T*_HB_ and *AV*_delay_, and is given directly as input neurons to the ANN. We stress that, differently from ref. ^23^, the initial reduced state vector **z**_0_ contains different sets of initial conditions for pressures, volumes and latent variables. Pressure and volume initial values are determined by model ${{{{\mathcal{M}}}}}_{3{{{\rm{D}}}}{{\mbox{-}}}0{{{\rm{D}}}}}$. Following^49^, latent variables are initialized to zero and these initial conditions act as additional tunable parameters along with the weights and biases of the ANN.

The loss function that we minimize during the ANN optimization process reads:

2 $$\begin{array}{ll}{{{\mathcal{L}}}}({{{\bf{z}}}}(t),{\tilde{\bf{z}}}(t);\widehat{{{{\bf{w}}}}})\,=\, \mathop{{\mathrm{arg}}\,{\mathrm{min}}}\limits_{\widehat{{{{\bf{w}}}}}}\left[\dfrac{| | {{{{\bf{z}}}}}_{{{{\rm{physical}}}}}(t)-{{\tilde{\bf{z}}}}_{{{{\rm{physical}}}}}(t)| {| }_{{{{{\rm{L}}}}}^{2}(0,{T}_{{{{\rm{HB}}}}})}^{2}}{{{{{\bf{z}}}}}_{{{{\rm{norm}}}}}^{2}}\right.\\ \qquad\qquad\qquad\qquad\,+\,\alpha \dfrac{{\left\Vert \dfrac{d{{{{\bf{z}}}}}_{{{{\rm{physical}}}}}(t)}{dt}-\dfrac{d{{\tilde{\bf{z}}}}_{{{{\rm{physical}}}}}(t)}{dt}\right\Vert }_{{{{{\rm{L}}}}}^{2}(0,{T}_{{{{\rm{HB}}}}})}^{2}}{{{{{\bf{z}}}}}_{{{{\rm{norm}}}},{{{\rm{diff}}}}}^{2}}\\ \qquad\qquad\qquad\qquad\,+\,\beta \dfrac{{\left(\mathop{\max}\limits_{t\in [0,{T}_{{{{\rm{HB}}}}}]}{{{{\bf{z}}}}}_{{{{\rm{physical}}}}}(t)-\mathop{\max}\limits_{t\in [0,{T}_{{{{\rm{HB}}}}}]}{{\tilde{\bf{z}}}}_{{{{\rm{physical}}}}}(t)\right)}^{2}}{{{{{\bf{z}}}}}_{{{{\rm{norm}}}},\max }^{2}}\\ \qquad\qquad\qquad\qquad\,+\,\gamma \dfrac{{\left(\mathop{\min}\limits_{t\in [0,{T}_{{{{\rm{HB}}}}}]}{{{{\bf{z}}}}}_{{{{\rm{physical}}}}}(t)-\mathop{\min}\limits_{t\in [0,{T}_{{{{\rm{HB}}}}}]}{{\tilde{\bf{z}}}}_{{{{\rm{physical}}}}}(t)\right)}^{2}}{{{{{\bf{z}}}}}_{{{{\rm{norm}}}},\min }^{2}}\\ \qquad\qquad\qquad\qquad\,+\,\eta \left(| | {{{{\bf{z}}}}}_{{{{\rm{latent}}}}}(0)| {| }^{2}+| | {{{{\bf{z}}}}}_{{{{\rm{latent}}}}}({T}_{{{{\rm{HB}}}}})| {| }^{2}\right)\\ \qquad\qquad\qquad\qquad\,\left.+\,\iota | | \widehat{{{{\bf{w}}}}}| {| }_{{{{{\rm{L}}}}}^{2}}^{2}\right],\end{array}$$

with *α* = *β* = *γ* = *η* = 0.1. The loss function aims at finding an optimal set of weights $\widehat{{{{\bf{w}}}}}$ for the ANN. It comprises the normalized mean square error between ANN pressure-volume predictions **z**_physical_(*t*) and observations ${\tilde{\bf{z}}}_{{{{\rm{physical}}}}}(t)$, as well as a weak penalization of the physical state vector time derivatives, maximum and minimum values for *t* ∈ [*T* − *T*_HB_, *T*]. Indeed, given the small ratio between the dimensionality of the training dataset and the number of parameters ***θ*** of model ${{{{\mathcal{M}}}}}_{3{{{\rm{D}}}}{{\mbox{-}}}0{{{\rm{D}}}}}$, we notice that these three additional terms reduce the generalization errors of the ANN. The penultimate weakly enforced condition on **z**_latent_(*t*) favors a periodic solution for all the hidden latent variables. The last term of the loss function prescribes the *L*^2^ regularization of the ANN weights and *ι* is one of the automatically tuned LNODEs hyperparameters (see Supplementary Material 4).

### Global sensitivity analysis

We employ the Saltelli’s method to perform a variance-based sensitivity analysis^50^. We compute both first-order Sobol indices and total-effect Sobol indices for each combination of quantity of interest and model parameter^36^. These two indices define how much varying a single parameter affects a specific QoI and how higher-order interactions among model parameters influences the model outputs, respectively. All mathematical details regarding the computation of Sobol indices and Saltelli’s sampling are given in Supplementary Material 5, along with first-order Sobol indices computed using model ${{{{\mathcal{M}}}}}_{{{{\rm{ANN}}}}}$.

### Robust parameter estimation

We perform parameter calibration with inverse UQ following a two-stage approach. First, given a set of time-dependent QoIs related to four-chamber heart pressure and volume traces, we solve a bounded and constrained optimization problem by employing model ${{{{\mathcal{M}}}}}_{{{{\rm{ANN}}}}}$ to obtain the pointwise MAP estimation for a predefined set of model parameters ${{{\boldsymbol{\theta }}}}\in {{{\boldsymbol{\Theta }}}}\subset {{\mathbb{R}}}^{{N}_{{{{\mathcal{P}}}}}}$. Second, we initialize HMC based on the MAP estimation and we build an approximation for the posterior distribution of ***θ***^37^, while accounting for the measurement and surrogate modeling errors via Gaussian Processes^24^. We provide all the mathematical and numerical details regarding the optimal control problem we solve, MAP estimation, HMC, and how we account for the different sources of error during inverse UQ in Supplementary Material 6.

### Software libraries

All 3D-0D closed-loop electromechanical simulations run with the Cardiac Arrhythmia Research Package (CARP)^13,51^. We train model ${{{{\mathcal{M}}}}}_{{{{\rm{ANN}}}}}$ by using an in-house high-performance Python library based on Tensorflow^52^. We perform GSA by means of the open source Python library SALib^53^. Parameter estimation with UQ is carried out by combining the open source Python libraries JAX^54^ and NumPyro^55^.

### Reporting summary

Further information on research design is available in the Nature Research Reporting Summary linked to this article.

### Supplementary information

Supplementary material (clean)

Reporting Summary

## Data Availability

The four-chamber heart pressure-volume loops used to train and test LNODEs are available at: https://github.com/MatteoSalvador/cardioEM-4CH.

## Code availability

The code to train LNODEs, to perform GSA and to do Bayesian parameter estimation with uncertainty quantification is publicly available at: https://github.com/MatteoSalvador/cardioEM-4CH.
