# Supplementary material for: Whole-heart electromechanical simulations using Latent Neural Ordinary Differential Equations
Source: NPJ Digit Med. 2024 Apr 11;7:90. doi: 10.1038/s41746-024-01084-x (PMC11009296; doi:10.1038/s41746-024-01084-x)
Supplement: Supplementary file 1 — Supplementary material (clean) [file 41746_2024_1084_MOESM1_ESM.pdf]

Supplementary Information

Whole-heart electromechanical simulations using Latent Neural Ordinary Differential Equations

Matteo Salvador[1,2,3,*], Marina Strocchi[2,4], Francesco Regazzoni[3], Christoph M. Augustin[5,6], Luca Dede'[3], Steven A. Niederer[2,4,7], Alfio Quarteroni[3,8]

[1] Institute for Computational and Mathematical Engineering, Stanford University, California, USA
[2] School of Biomedical Engineering and Imaging Sciences, King's College London, London, UK
[3] MOX, Department of Mathematics, Politecnico di Milano, Milan, Italy
[4] National Heart and Lung Institute, Imperial College London, London, UK
[5] Institute of Biophysics, Medical University of Graz, Graz, Austria
[6] BioTechMed-Graz, Graz, Austria
[7] The Alan Turing Institute, London, UK
[8] École Polytechnique Fédérale de Lausanne, Lausanne, Switzerland (*Professor Emeritus*)

[*] *Corresponding author* (`msalvad@stanford.edu`)

Supplementary Material 1: Four-chamber heart geometry

The cardiac geometry comes from a 77 yo female heart failure patient with reduced ejection fraction (smaller than 35% for the left ventricle), an indication for cardiac resynchronization therapy, and atrial fibrillation. The left ventricular ejection fraction simulated by the model was representative of this cohort ($31\% \pm 4\%$, ranging between 22% and 38%). The left ventricular end-diastolic pressure in this patient was low (2.8 mmHg), potentially due to atrial fibrillation, and the one simulated by the model (5.8 ± 1.61 mmHg, ranging from 2.4 mmHg to 11.26 mmHg) fell within literature data (9 ± 3 mmHg) of atrial fibrillation patient during sinus rhythm [53]. The average wall thickness of the left ventricle was 6 mm, which is in range with left ventricular wall thickness measured in healthy female controls [58].

The end-diastolic computed tomography (CT) image acquired from this female heart failure patient was segmented to generate a four-chamber heart geometry. All the computational tools regarding segmentation and meshing with 1 mm linear tetrahedral Finite Elements are described in [51, 52]. The atria are refined with the resample algorithm from meshtool [36] to have at least 3 elements across the wall thickness to reduce locking effects. The ventricles were assigned with a transmural fibre distribution using the Bayer's rule-based algorithm [6] (Figure 1, bottom right), where the fibre and sheet angles at the endocardium and epicardium are $+60^\circ$ and -60° [40], and -65° and $+25^\circ$ [6], respectively. Atrial myofibre orientation was assigned by computing universal atrial coordinates on the atria and by mapping an ex-vivo diffusion tensor MRI dataset onto the endocardial and the epicardial surfaces (Figure 1, top right) [23, 45]. The transmural fibre orientation was set to be the endocardial and the epicardial orientation for elements below and above 50% of the wall thickness, respectively. We refer to [53] for further details about this patient-specific geometry.

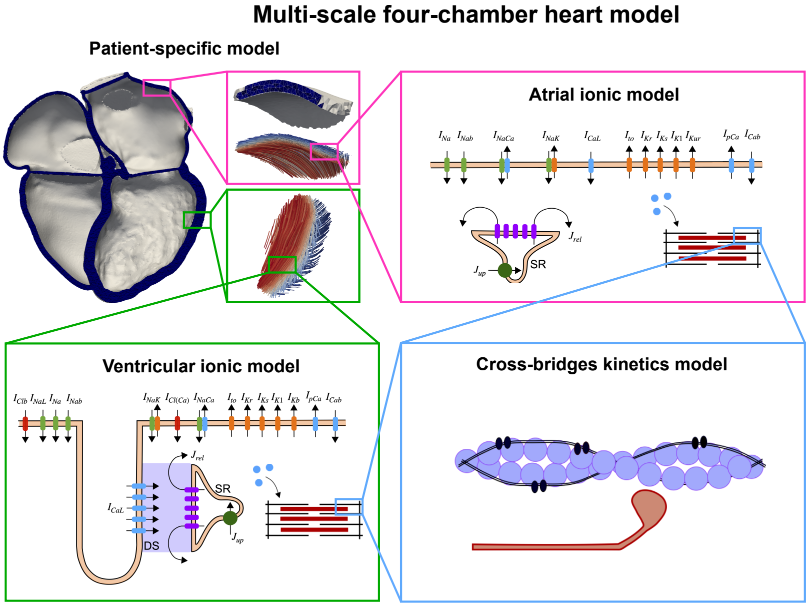

Supplementary Figure 1: Multi-scale four-chamber heart model. Patient-specific whole-heart with refined atrial and ventricular transmural myofiber orientation (top left), atrial (top right) and ventricular (bottom left) ionic model, cross-bridges dynamics (bottom right) [53].

Supplementary Material 2: Mathematical and numerical modeling of the 3D-0D solver

Let $\Omega \subset \mathbb{R}^3$ be the domain corresponding to the patient-specific four-chamber heart. We employ the reaction-Eikonal model without diffusion for cardiac electrophysiology [35]. We report the Eikonal model in Equation (1). Given $\mathbf{V}(\mathbf{x})$ containing the squared local conduction velocities (CV) in the fibres, sheet and normal to sheet directions, and sites of initial activation Γ, this equation allows to find the local activation times $t_a(\mathbf{x})$ at node location $\mathbf{x}$, with initial activation occurring at a prescribed time t_0:

$$\begin{cases} \sqrt{\nabla t_a(\mathbf{x})^T \mathbf{V}(\mathbf{x}) \nabla t_a(\mathbf{x})} = 1 & \mathbf{x} \in \Omega, \\ t_a(\mathbf{x}) = t_0 & \mathbf{x} \in \Gamma. \end{cases} \tag{1}$$

We represent atria and ventricles as transversely isotropic conductive regions. In particular, we assign CVs in the fibre direction ($\mathrm{CV}_{\mathrm{f,V}}$ and $\mathrm{CV}_{\mathrm{f,A}}$) and anisotropy ratios ($\mathrm{k}_{\mathrm{ft,V}}$ and $\mathrm{k}_{\mathrm{ft,A}}$), respectively. The remaining regions are considered as passive. To represent fast endocardial activation due to the His–Purkinje system, we introduce a 1-mm element thick endocardial layer extending up to 70% in the apico-basal direction of the ventricles [26, 51], with faster CV compared to the rest of ventricular myocardium of a factor k_{FEC} (Figure 2, right). We account for the Bachmann bundle by defining a region between the left atrium (LA) and the right atrium (RA) with fast CV compared to the rest of the atrial myocardium of a factor k_{BB} (Figure 2, left) [45]. To fully control the atrioventricular (AV) delay, we define a passive region along the AV plane to insulate the atria

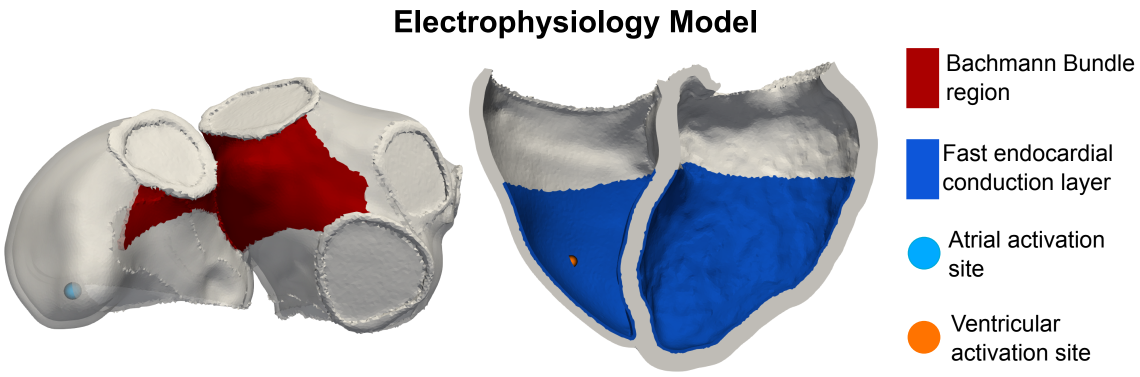

Supplementary Figure 2: Electrophysiology simulations. Atria with the region representing the Bachmann bundle and the atrial activation site (left). Ventricles with the fast endocardial conduction layer and the ventricular activation site (right) [53].

from the ventricles. Atrial activation is triggered at the location of the RA lead, while ventricular activation is initiated at the RV lead location with a delay defined by the AV delay, included as a free parameter in the simulator (AV_{delay}). The RA and RV lead locations were selected by segmenting the pacemaker leads from the CT image by thresholding the image intensity.

We employ the Courtemanche-Ramirez-Nattel (CRN) [10] ionic model for atrial cardiomyocytes and the Tomek-Rodriguez-O'Hara-Rudy (ToR-ORd) ionic model with a dynamic representation of intracellular chloride [54, 55] for ventricular cardiomyocytes, respectively. We induce the initial increase in the transmembrane potential V_{m} by imposing a foot current that acts as a local stimulus, activating the cell membrane in each point $\mathbf{x}$ of the domain Ω at the local activation time $t_a(\mathbf{x})$ computed with the Eikonal model [35].

The intracellular calcium transient obtained from the ionic model is provided as an input to the Land contraction model [25] to compute the active tension transient in atria and ventricles. For the sake of simplicity, we assume that active contraction occurs in the fibre direction only. Prior to the 3D-0D closed-loop electromechanical simulations, the ToR-ORd-Land and CRN-Land cell models were run for 500 heartbeats at a basic cycle length of 0.854 s, which corresponds to the heartbeat period T_{HB} of the patient, to reach a steady state.

We use the transversely isotropic Guccione model for atrial and ventricular passive mechanics [16], according to which the strain energy function takes the following expression:

$$\begin{aligned}\Psi(\mathbf{E}) &= \frac{a}{2}\left[e^{Q}-1\right]+\frac{\kappa}{2}\left(\log J\right)^{2} \\ Q &= b_{\mathrm{f}}E_{\mathrm{ff}}^{2}+2b_{\mathrm{ft}}(E_{\mathrm{fs}}^{2}+E_{\mathrm{fn}}^{2})+b_{\mathrm{t}}(E_{\mathrm{ss}}^{2}+E_{\mathrm{nn}}^{2}+2E_{\mathrm{sn}}^{2})\ ,\end{aligned} \tag{2}$$

where J is the determinant of the deformation tensor, $\mathbf{E}$ represents the Green-Lagrange strain tensor and f, s and n are the fibre, sheet and normal to sheet directions. a, b_{f}, b_{ft} and b_{t} are the stiffness parameters, whereas $\kappa = 1000$ kPa is the bulk modulus, penalising volume changes and therefore enforcing quasi-incompressibility [13, 38]. The Guccione model is largely employed in recent papers showing four-chamber heart electromechanical simulations [5, 12, 14, 53]. Moreover, it contains less parameters than more sophisticated alternatives [17, 27, 37], which makes it amenable to patient-specific calibration while still capturing the behavior of a transversely isotropic material. Passive material properties of all the other cardiac tissues are represented by means of a Neo-Hookean model,

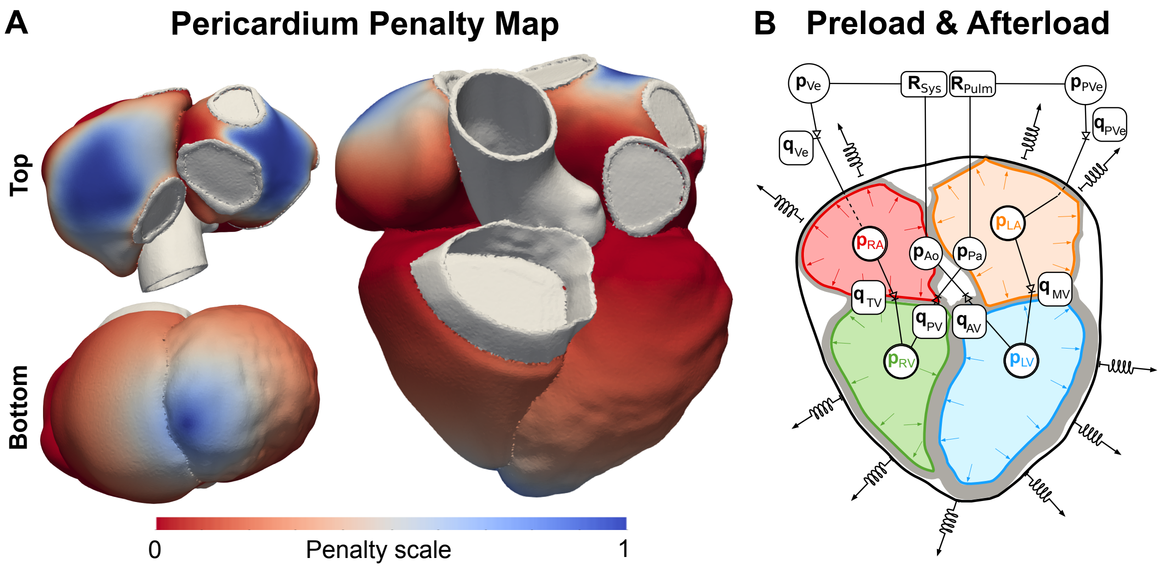

Supplementary Figure 3: Boundary conditions for the mechanical model. **A** Penalty map scaling the normal spring stiffness for the effect of the pericardium. **B** Afterload and preload boundary conditions represented with CircAdapt. Symbols and abbreviations: **p**=pressure, **R**=resistance, **q**=flow across a valve, LV=left ventricle, RV=right ventricle, LA=left atrium, RA=right atrium, Ao=aorta, Pa=pulmonary artery, Ve=veins, PVe=pulmonary veins, sys=systemic, pulm=pulmonary, MV=mitral valve, TV=tricuspid valve, AV=aortic valve, PV=pulmonary valve [53].

with the stiffness parameters following previous studies [51, 52].

As described in [5, 51], we simulate the pericardium effect on the heart with normal springs with stiffness k_{peri}. This value is scaled on the ventricles according to a map derived from motion data [52], to constrain the motion of the apex but not the base, allowing for physiological AV plane downward displacement during ventricular systole. A similar analysis on the atria, described in [50], showed that the roof of the atria moved the least, while the regions around the AV plane moved the most, as they are stretched down by the contracting ventricles. We therefore define a scaling map on the atria to include this constraint in the model, by assigning maximum penalty to the roof of the atria and zero penalty towards the AV plane (Figure 3A). In addition, we apply omni-directional springs to the right inferior and superior pulmonary veins and at the superior vena cava rings. The stiffness of these springs is fixed to 1.0 kPa/μm [53].

The 3D four-chamber electromechanical model is coupled with the 0D closed-loop CircAdapt model [4, 57] (Figure 3B), which represents the following components of the circulatory system: aorta, pulmonary artery, veins, systemic and pulmonary peripheral resistances, the four cardiac valves (aortic, pulmonary, mitral and tricuspid) and flows across the pulmonary veins into the LA and across the systemic veins into the RA. The monolithic 3D-0D coupling method is described in [5]. Briefly, the pressures of the LA, LV, RA and RV were included as additional unknowns to the monolithic scheme, and the following equations are added to the equations of passive mechanics:

$$
\begin{aligned}
V_{\mathrm{LV}}^{\mathrm{3D}}(\mathbf{u},t) - V^{\mathrm{0D}}(p_{\mathrm{LV}},t) &= 0 \ , \\
V_{\mathrm{RV}}^{\mathrm{3D}}(\mathbf{u},t) - V^{\mathrm{0D}}(p_{\mathrm{RV}},t) &= 0 \ , \\
V_{\mathrm{LA}}^{\mathrm{3D}}(\mathbf{u},t) - V^{\mathrm{0D}}(p_{\mathrm{LA}},t) &= 0 \ , \\
V_{\mathrm{RA}}^{\mathrm{3D}}(\mathbf{u},t) - V^{\mathrm{0D}}(p_{\mathrm{RA}},t) &= 0 \ ,
\end{aligned}
$$

where V^{3D} and V^{0D} are the volumes of the cavity computed from the deforming 3D mesh and predicted by the 0D model, respectively, t is the time and $\mathbf{u}$ is the displacement field. The evolution of V^{3D} and V^{0D} in the different cardiac chambers is defined by the 3D electromechanical model and the 0D closed-loop CircAdapt circulation model, respectively. At each time step, the values of p_{LV}, p_{RV}, p_{LA} and p_{RA} are determined by enforcing these four volumetric constraints. This overall defines the simulated pressure-volume relationship of the patient.

The ventricles of the end-diastolic mesh are unloaded from an end-diastolic LV and RV pressure, while the atria are not unloaded, under the assumption that the active tension in the atrial myocardium balances the pressure [25]. During the unloading phase, we do not apply pericardial boundary conditions at the epicardium. Then, prior to the start of the 3D-0D coupled simulation, we reloaded the ventricles to retrieve the end-diastolic mesh while the atrial pressure is initialised at 0 mmHg. The electromechanical simulations always start at end-diastole and the pericardial boundary conditions are actived in this phase. We remark that, differently from [53], the end-diastolic pressures do not act as additional model parameters but are instead prescribed as initial conditions for the system of LNODEs.

To minimise the effect of these initial conditions, we run all numerical simulations for 5 heartbeats, to reach a near-to-steady-state behaviour, on a supercomputer endowed with 512 cores. Figures 4 and 5 show the pressure-volume dynamics of all the $N_{\mathrm{sims}} = 405$ electromechanical simulations considered for training, validation and testing phases. Given the significant amount of required computational power, we select the linear and non-linear solver relative tolerances for passive mechanics in such a way to reduce the overall computational time while preserving accuracy [53]. In particular we set the maximum number of Newton iterations to 1 for the first three heartbeats. Indeed, as shown in [5], this approach brings the numerical simulation closer to a steady state before solving nonlinear passive mechanics more accurately with more Newton iterations. As a matter of fact, we set the maximum number of Newton iterations to 2 for the last two heartbeats in order to have a better approximation of the stretch rate for the cell model. We also increase the tolerance for the numerical solution of the linearised system to 10^{-4}, for all heartbeats. We show in [53] that these numerical settings have limited effects on the pressure-volume dynamics simulated by the 3D-0D closed-loop electromechanical model while allowing for a 3 times speed-up in the total computational time. We refer to [53] for further details about the mathematical and numerical model.

Supplementary Material 3: Model parameters

In Table 1, we report the list of parameters covering the whole cardiac and cardiovascular function that has been used to train the system of LNODEs. The choice of the specific parameter values and their ranges is motivated by the comprehensive study performed in [53], where Strocchi et al. train several Gaussian Processes Emulators to carry out global sensitivity analysis and history matching. In particular, starting from 117 model parameters of interest, this technique allows to exclude unimportant ones, whereas the latter permits to identify implausible areas that would provide unphysiological outputs. In Strocchi et al., the transition from 117 to 43 model parameters

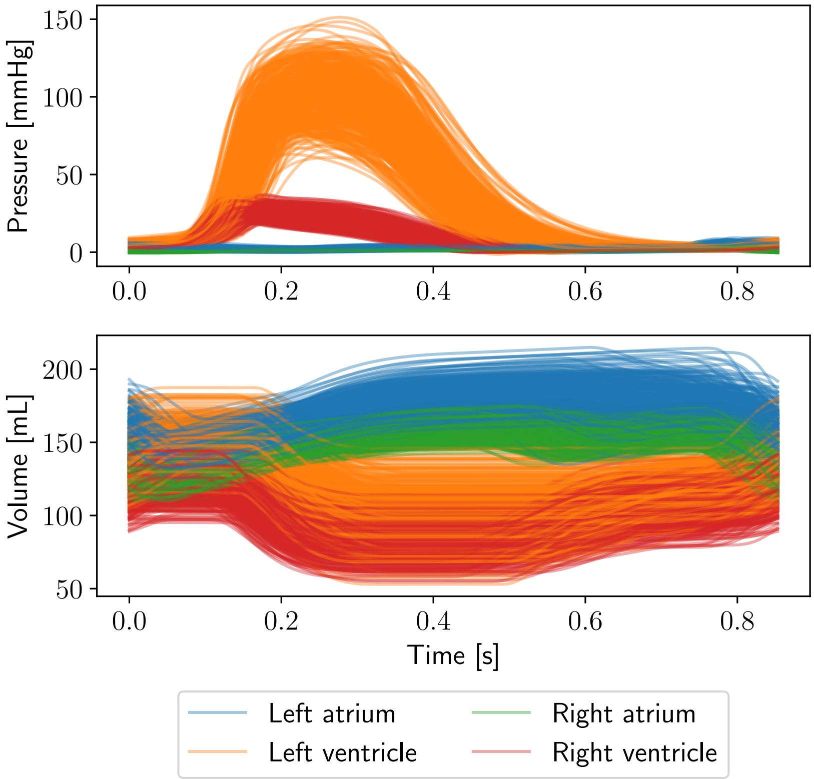

Supplementary Figure 4: Pressure and volume time traces of the $N_{\text{sims}} = 405$ electromechanical simulations considered for training, validation and testing phases. We run the numerical simulations for 5 heartbeats (heartbeat period $T_{\text{HB}} = 0.854$ s) and then we take the last cardiac cycle.

has been determined by looking at phenotypes across different scales and physics (action potential, calcium and tension transient, activation times, pressure and volume). In this way, we can ascertain that we did not miss any relevant parameters for the pressure-volume relationship, as this was considered in Strocchi et al. among the different phenotypes. The geometry-independent model parameters were kept general, as the goal of the study was to demonstrate the validity of a generic, rather than heart failure-specific, framework. For instance, at the cellular level, the mathematical models were constrained to mimic a wide range of plausible calcium transients potentially applicable to different patients. We refer to the Supplementary Information of Strocchi et al. for a detailed description of how the global sensitivity analysis has been performed on the different sub-models of cardiac electromechanics.

In Figure 6, we show the distribution of some relevant model parameters according to global sensitivity analysis. In particular, we choose the subset corresponding to the $\mathcal{T}_{\text{ventricles}}$ test case. We notice that the testing samples do not overlap with the training ones, and in some cases they lie in areas of the parameter space that are not well explored during the training stage of LNODEs (see for instance the subplots ventricular steady-state duty ratio $dr^{\text{ToRORd-Land}}$ vs. ventricular

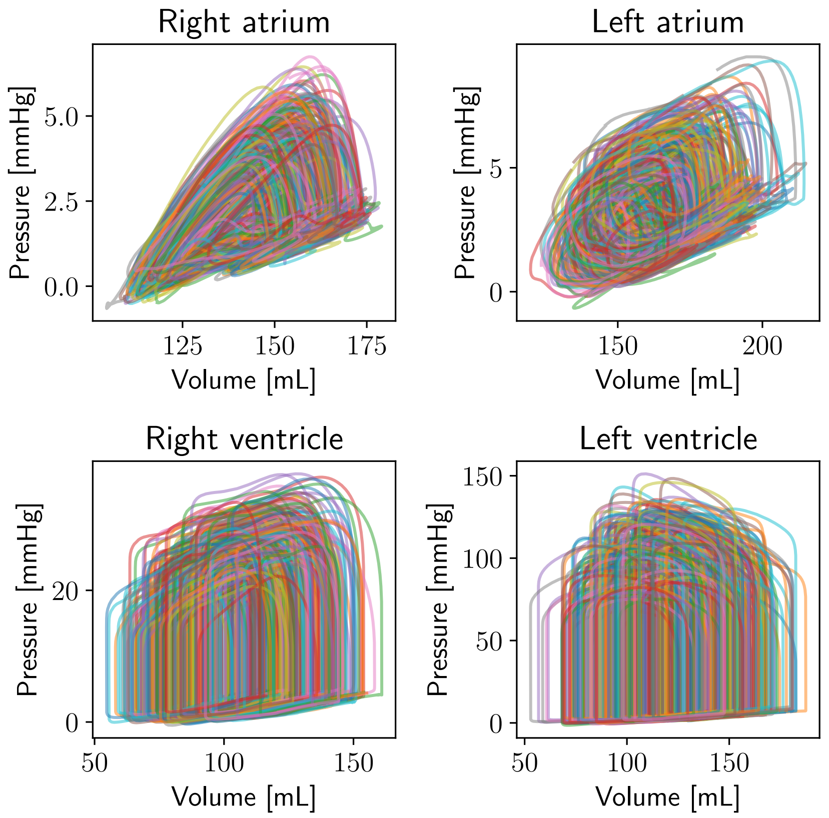

Supplementary Figure 5: Corresponding pressure-volume loops of the $N_{\mathrm{sims}} = 405$ electromechanical simulations used for training, validation and testing phases.

calcium/troponin complex when 50% of crossbridges are blocked $perm_{50}^{\mathrm{ToRORd-Land}}$ and ventricular calcium/troponin complex when 50% of crossbridges are blocked $perm_{50}^{\mathrm{ToRORd-Land}}$ vs. systemic resistance scaling factor R^{sys}). Nevertheless, by looking at the main text (Table 1), we see that the trained system of LNODEs show small testing errors, ranging from 2% to 6%.

Supplementary Material 4: Training of the Latent Neural Ordinary Differential Equations

We perform hyperparameters tuning by employing K-fold ($K = 10$) cross validation over 400 electromechanical simulations. An optimal set of hyperparameters is automatically found by running the Tree-structured Parzen Estimator (TPE) Bayesian algorithm [3, 7] while monitoring the generalization error reported in the main text (Section 2.1, Equation (2)) during K-fold cross validation. We early stop bad hyperparameters configurations by means of the Asynchronous Successive Halving (ASHA) scheduler [28, 29]. We rely on the Ray Python distributed framework for the implementation of this hyperparameters tuner [32]. Different ANNs associated to different hyperparameters settings

Parameter	Description	Range	Refs.
$I_{\text{up}}^{\text{max,CRN}}$	AT maximum Ca^{2+} uptake rate into the sarcoplasmic reticulum network	[0.0028, 0.0080]	[10]
$TRPN^{\text{max,CRN}}$	AT total troponin C concentration in cytoplasm	[0.0359, 0.1039]	[10]
$g_{\text{CaL}}^{\text{CRN}}$	AT conductance of L-type Ca^{2+} current	[0.08910, 0.1979]	[10]
$T_{\text{ref}}^{\text{CRN-Land}}$	AT reference isometric tension	[80.1142, 119.934]	[25]
$perm_{50}^{\text{CRN-Land}}$	AT calcium/troponin complex when 50% of crossbridges are blocked	[0.1802, 0.5233]	[25]
$nperm^{\text{CRN-Land}}$	AT Hill coefficient for Ca^{2+}-troponin and unbound sites	[2.54302, 7.3862]	[25]
$TRPN_{\text{n}}^{\text{CRN-Land}}$	AT Ca^{2+}-troponin cooperativity	[1.0222, 2.9841]	[25]
$dr^{\text{CRN-Land}}$	AT steady-state duty ratio	[0.1271, 0.3711]	[25]
$w_{\text{frac}}^{\text{CRN-Land}}$	AT steady-state ratio between weakly and strongly bound sites	[0.2612, 0.7461]	[25]
$TOT_{\text{A}}^{\text{CRN-Land}}$	AT scale for distortion due to velocity of contraction	[12.7021, 37.1894]	[25]
$\phi^{\text{CRN-Land}}$	AT distortion decay	[1.1738, 3.4305]	[25]
$ca_{50}^{\text{CRN-Land}}$	AT reference Ca^{2+} sensitivity	[0.5678, 1.2880]	[25]
$mu^{\text{CRN-Land}}$	AT scaling factor for weakly to strongly transition rate	[4.6065, 13.3936]	[25]
CV^{atria}	Atrial conduction velocity in the fibre direction	[0.7508, 1.0269]	[53]
k_{BB}	Bachmann bundle scaling factor	[1.7011, 5.6372]	[53]
a^{atria}	AT bulk myocardium stiffness	[1.5095, 2.4999]	[24, 39]
$b_{\text{f}}^{\text{atria}}$	AT stiffness in the fibre direction	[4.0493, 11.9966]	[24, 34]
$b_{\text{t}}^{\text{atria}}$	AT stiffness in the transverse plane	[1.5192, 4.4989]	[24, 34]
$PCa_{\text{b}}^{\text{ToRORd}}$	VE conductance of the background Ca^{2+} current	[6.3660e-05, 1e-04]	[54]
$TRPN^{\text{max,ToRORd}}$	VE maximum troponin C concentration	[0.065, 0.1228]	[54]
$GNCX_{\text{b}}^{\text{ToRORd}}$	VE conductance of the Na^{2+}-Ca^{2+} exchanger	[0.0009, 0.0024]	[54]
$T_{\text{ref}}^{\text{ToRORd-Land}}$	VE reference isometric tension	[127.846, 199.575]	[25]
$perm_{50}^{\text{ToRORd-Land}}$	VE calcium/troponin complex when 50% of crossbridges are blocked	[0.1764, 0.5117]	[25]
$nperm^{\text{ToRORd-Land}}$	VE Hill coefficient for Ca^{2+}-troponin and unbound sites	[1.8542, 3.0441]	[25]
$TRPN_{\text{n}}^{\text{ToRORd-Land}}$	VE Ca^{2+}-troponin cooperativity	[1.8390, 2.9980]	[25]
$dr^{\text{ToRORd-Land}}$	VE steady-state duty ratio	[0.1263, 0.3627]	[25]
$w_{\text{frac}}^{\text{ToRORd-Land}}$	VE steady-state ratio between weakly and strongly bound sites	[0.2884, 0.7462]	[25]
$TOT_{\text{A}}^{\text{ToRORd-Land}}$	VE scale for distortion due to velocity of contraction	[12.6888, 37.392]	[25]
$ktm_{\text{unblock}}^{\text{ToRORd-Land}}$	VE transition rate from blocked to unblocked binding site	[0.0123, 0.0315]	[25]
$ca_{50}^{\text{ToRORd-Land}}$	VE reference Ca^{2+} sensitivity	[0.4071, 1.0490]	[25]
$mu^{\text{ToRORd-Land}}$	VE scaling factor for weakly to strongly transition	[1.5216, 4.4844]	[25]
$CV^{\text{ventricles}}$	VE conduction velocity in the fibre direction	[0.3832, 0.7967]	[53]
k_{FEC}	Fast endocardial layer scaling factor	[1.3250, 8.3687]	[53]
$a^{\text{ventricles}}$	VE bulk myocardium stiffness	[0.5006, 1.4998]	[24]
$b_{\text{t}}^{\text{ventricles}}$	VE stiffness in the transverse plane	[1.5042, 4.49251]	[24, 34]
a^{lvrv}	Scaling factor for $a^{\text{ventricles}}$ in RV vs. LV	[1.0055, 1.9995]	[39]
$T_{\text{ref}}^{\text{lvrv}}$	Scaling factor for $T_{\text{ref}}^{\text{ToRORd-Land}}$ in RV vs. LV	[0.5009, 0.9956]	[39]
AV_{delay}	Atrioventricular delay	[0.1, 0.2]	[20]
k_{peri}	Pericardial normal springs stiffness	[0.0005, 0.0019]	[52]
R^{sys}	Systemic resistance scaling factor	[1.0017, 3.9937]	[5, 57]
R^{pulm}	Pulmonary resistance scaling factor	[1.0020, 3.9980]	[5, 57]
Aol	Length of the aorta	[300.478, 498.745]	[5, 57]
k^{Art}	Stiffness of the aorta	[6.0118, 9.9894]	[5, 57]

Supplementary Table 1: Parameter space explored by model $\mathcal{M}_{\text{3D-0D}}$ and used for the ANN training. From top to bottom: atria, ventricles, whole-heart, circulation. AT: atrial, VE: ventricular.

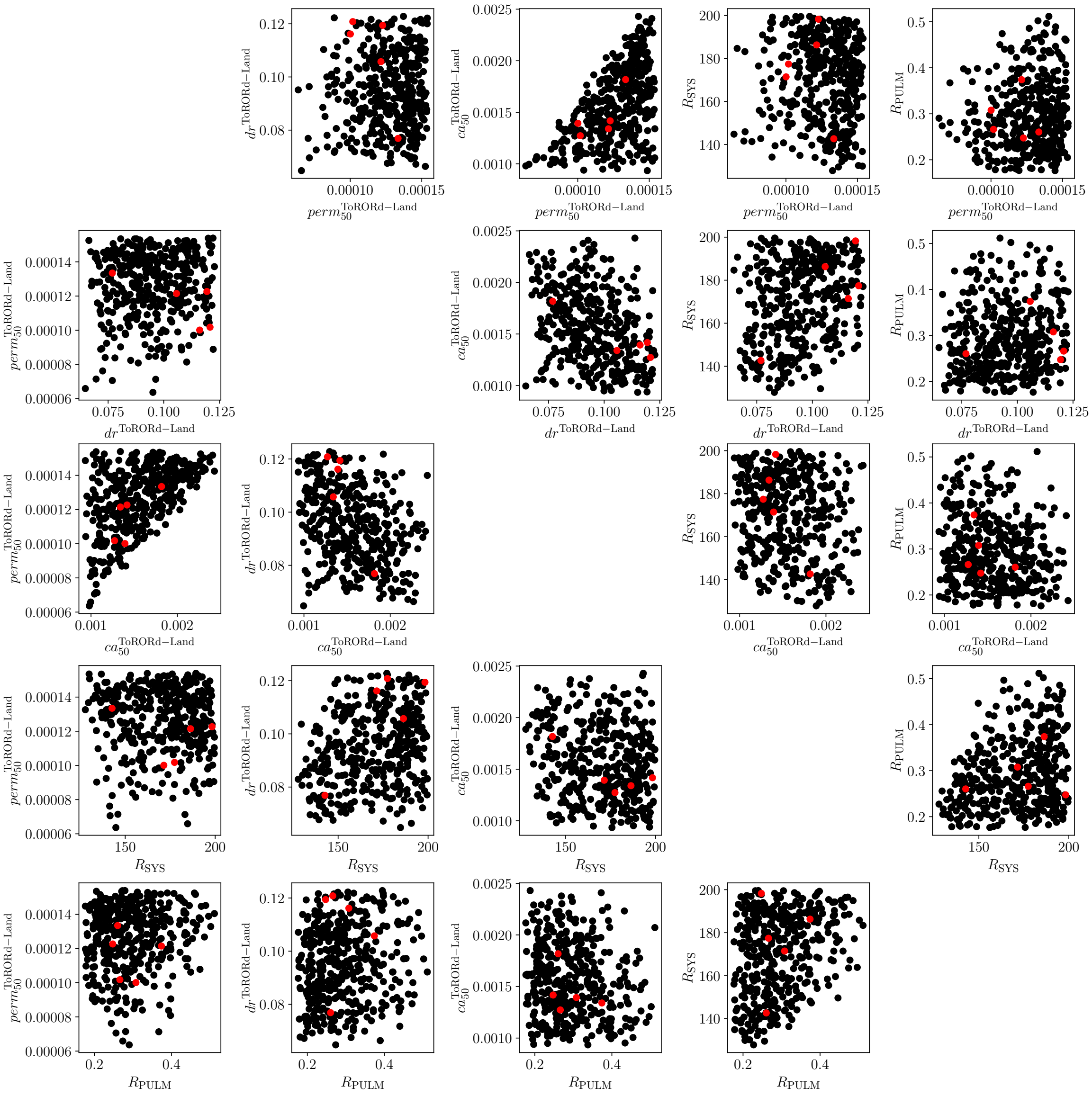

Supplementary Figure 6: Distribution of training (black) and testing (red) samples in the parameter space using the most relevant model parameters employed in the $\mathcal{T}_{\text{ventricles}}$ test case.

are simultaneously trained with Message Passing Interface (MPI) on 40 cores of a high-performance computing facility at MOX, Dipartimento di Matematica, Politecnico di Milano. We also exploit Hyper-Threading via Open Multi-Processing (OpenMP) to speed-up tensorial operations in Tensorflow [1]. We consider an hypercube as a search space for the following hyperparameters: number of layers and neurons of the ANN, number of states N_z, loss function $\mathcal{L}(\mathbf{z}(t), \widetilde{\mathbf{z}}(t); \widehat{\mathbf{w}})$ integration step Δt_{ref} and L^2 weights regularization ι. For each configuration of hyperparameters, we perform 1'000 iterations with the first-order Adam optimizer [21], starting with a learning rate of 10^{-2}, and

LNODE	Hyperparameters					Trainable parameters
	layers	neurons	num. states	loss integr. step [s]	weights reg.	# param.
tuning	$\{1 \ldots 7\}$	$\{5 \ldots 50\}$	$\{8 \ldots 12\}$	$[10^{-3}, 0.1]$	$[10^{-4}, 1]$	
final	3	13	8	0.0285	0.023	1'178

Supplementary Table 2: Hyperparameters ranges and selected values for the final training stage of LNODEs.

then we continue the training stage with 10'000 iterations of the second-order BFGS optimizer [15]. In this way, we exploit the stochastic behavior of the Adam optimizer to explore the landscape of local minima, and then we properly reach convergence by means of the BFGS optimizer. The ANN is always initialized with a new set of weights provided by a Glorot uniform distribution and zero values for biases. In Table 2, we report the initial hyperparameters ranges for tuning and the final optimized values.

Supplementary Material 5: Global sensitivity analysis

To assess how much each model parameter $\boldsymbol{\theta}_i$ affects a pressure-volume biomarker of clinical interest for the atrial or ventricular function, that is a QoI $\mathbf{q}_j$, we perform a global sensitivity analysis. This is typically done by sampling the parameter space and by computing suitable indicators. Relevant choices of these indicators are given by Borgonovo indices [41], Sobol indices [19, 48], Morris elementary effects [33], Shapley values [49], ANCOVA indices [59] and Kucherenko indices [22].

In this work, we perform a variance-based sensitivity analysis, which relies on a probabilistic approach, by computing Sobol indices [48]. The first-order Sobol index S_i^j evaluates the impact that a single parameter $\boldsymbol{\theta}_i$ has on a certain QoI $\mathbf{q}_j$, whereas the total-effect Sobol index $S_i^{j,T}$ also accounts for the interactions among parameters:

$$S_i^j = \frac{\mathbb{V}\mathrm{ar}_{\boldsymbol{\theta}_i}\left[\mathbb{E}_{\boldsymbol{\theta}_{\sim i}}\left[\mathbf{q}_j|\boldsymbol{\theta}_i\right]\right]}{\mathbb{V}\mathrm{ar}\left[\mathbf{q}_j\right]}, \qquad S_i^{j,T} = \frac{\mathbb{E}_{\boldsymbol{\theta}_{\sim i}}\left[\mathbb{V}\mathrm{ar}_{\boldsymbol{\theta}_i}\left[\mathbf{q}_j|\boldsymbol{\theta}_{\sim i}\right]\right]}{\mathbb{V}\mathrm{ar}\left[\mathbf{q}_j\right]} = 1 - \frac{\mathbb{V}\mathrm{ar}_{\boldsymbol{\theta}_{\sim i}}\left[\mathbb{E}_{\boldsymbol{\theta}_i}\left[\mathbf{q}_j|\boldsymbol{\theta}_{\sim i}\right]\right]}{\mathbb{V}\mathrm{ar}\left[\mathbf{q}_j\right]},$$

where $\boldsymbol{\theta}_{\sim i}$ indicates the set of all parameters excluding the ith one.

We employ the Saltelli's method and model $\mathcal{M}_{\mathrm{ANN}}$ to estimate these Sobol indices [19, 46]. This allows for a linear increase in the number of samples $N_{\mathcal{S}}$ with respect to the number of parameters $N_{\mathcal{P}}$ once a certain accuracy is prescribed. Specifically, the number of samples $N_{\mathcal{S}}$ in the parameter space scales as $N(2N_{\mathcal{P}}+2)$, being N a user defined value. In this work, we set $N = 8000$, for a total of 704'000 samples, that allows for small confidence intervals around the first-order and total-effect Sobol indices, respectively.

The model parameters may arbitrarily vary in the training ranges defined in Table 1. The QoIs are given by the maximum and minimum values of the four-chamber pressures, volumes and corresponding time derivatives of the simulated heartbeat with the trained LNODEs. We employ the forward Euler method with a fixed time step $\Delta t = 10^{-3}$ s for all the numerical simulations with model $\mathcal{M}_{\mathrm{ANN}}$.

We report first-order Sobol indices in Figure 7. By comparing them to total-effect Sobol indices in Figure 3 from the main text, we notice that there are some significant differences. In particular, high-order interactions among model parameters are important, as the difference between total-effect and first-order Sobol indices is higher than 0.05 for many relevant cases. This holds

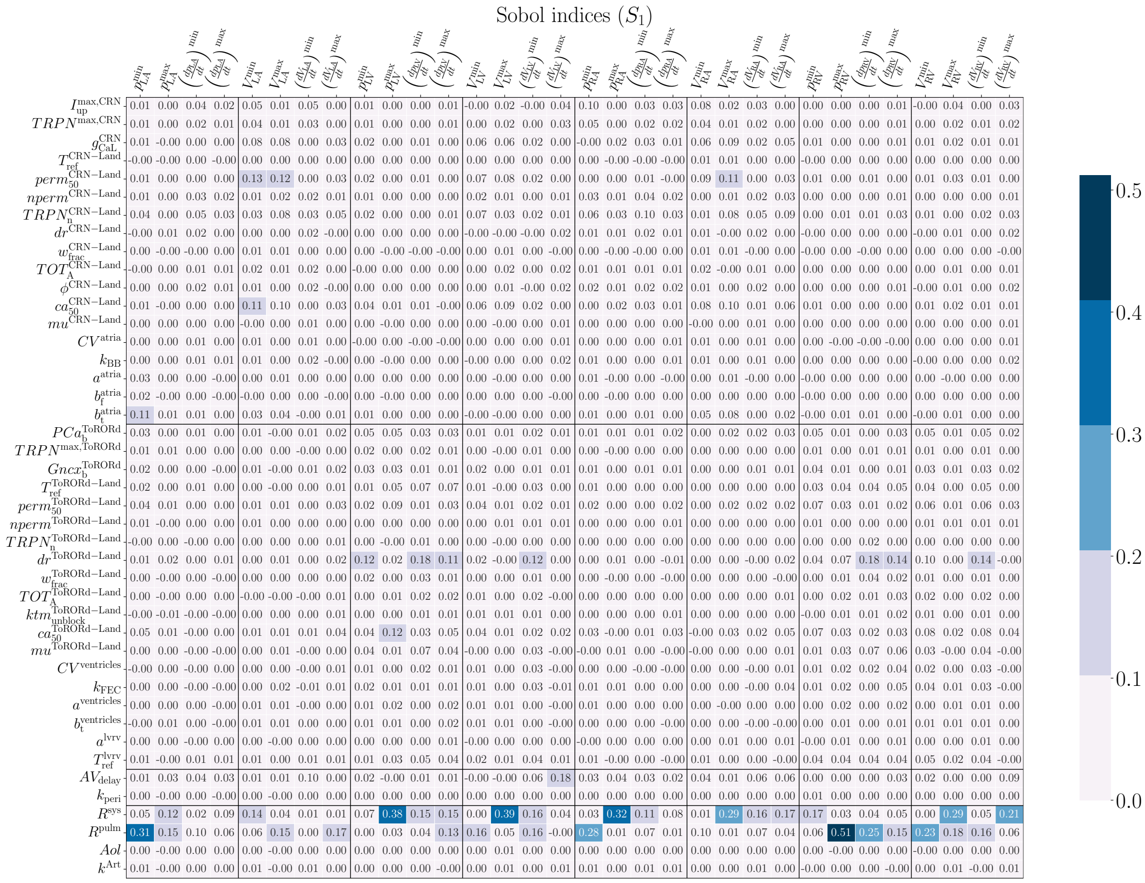

Supplementary Figure 7: First-order Sobol indices S_i^j computed by exploiting model $\mathcal{M}_{\text{ANN}}$. Each row corresponds to a parameter $\boldsymbol{\theta}$ of the 3D-0D closed-loop electromechanical model (see Table 1). Each column corresponds to a QoI $\mathbf{q}$ (maximum and minimum of the temporal traces of pressures or volumes and of their time derivatives). Both parameters and QoIs are split into a number of groups, separated by a black solid line. Specifically, from left to right, we list QoIs referred to LA, LV, RA and RV. From top to bottom, we list model parameters associated with atria, ventricles, whole-heart dynamics and cardiovascular system.

for most of the influential parameters coming from the CRN-Land model, such as atrial conductance of L-type Ca^{2+} current $g_{\mathrm{CaL}}^{\mathrm{CRN}}$, atrial calcium/troponin complex when 50% of crossbridges are blocked $perm_{50}^{\mathrm{CRN-Land}}$, atrial Ca^{2+}-troponin cooperativity $TRPN_{\mathrm{n}}^{\mathrm{CRN-Land}}$ and atrial reference Ca^{2+} sensitivity $ca_{50}^{\mathrm{CRN-Land}}$, as well as driven from the ToRORd-Land model, such as ventricular calcium/troponin complex when 50% of crossbridges are blocked $perm_{50}^{\mathrm{ToRORd-Land}}$, ventricular reference isometric tension $T_{\mathrm{ref}}^{\mathrm{ToRORd-Land}}$ and ventricular reference Ca^{2+} sensitivity $ca_{50}^{\mathrm{ToRORd-Land}}$. Similar considerations can be done for other ventricular (ventricular conduction velocity in the fibre direction $CV^{\mathrm{ventricles}}$, fast endocardial layer scaling factor k_{FEC}) and cardiovascular parameters (systemic resistance scaling factor R^{sys}, pulmonary resistance scaling factor R^{pulm}) with respect to specific QoIs. As in the case with total-effect Sobol indices, we notice that the QoIs associated with a given area of the cardiovascular system are still mostly determined by the parameters associated with the same region. However, similar important exceptions can be outlined, which is the case for the systemic and pulmonary resistances (R^{sys}, R^{pulm}), which explain variability for the whole-heart. The AV delay AV_{delay} has a major impact on almost all biomarkers and can be inferred from the patient-specific volume traces over time without the need for parameter calibration.

Supplementary Material 6: Robust parameter estimation

Let $\boldsymbol{\theta} \in \boldsymbol{\Theta} \subset \mathbb{R}^{N_{\mathcal{P}}}$ be a subset of parameters for the model $\mathcal{M}_{\text{3D-0D}}$ that we want to calibrate with model $\mathcal{M}_{\text{ANN}}$, when certain time-dependent QoIs are provided as observations. We carry out the Maximum a Posteriori (MAP) estimation by solving a constrained optimal control problem [47]:

$$\min_{\boldsymbol{\theta} \in \boldsymbol{\Theta}} J(\boldsymbol{\theta}), \tag{3}$$

with the following cost functional:

$$\begin{aligned} J(\boldsymbol{\theta}) = & \sum_{\mathrm{i} \in \{\text{LA, LV, RA, RV}\}} \epsilon_{\mathrm{i}} \frac{||p_{\mathrm{i}}(t;\boldsymbol{\theta}) - \hat{p}_{\mathrm{i}}(t)||^2_{\mathrm{L}^2}}{\mu_{p_{\mathrm{i}}^2}} \\ + & \sum_{\mathrm{i} \in \{\text{LA, LV, RA, RV}\}} \zeta_{\mathrm{i}} \frac{||V_{\mathrm{i}}(t;\boldsymbol{\theta}) - \hat{V}_{\mathrm{i}}(t)||^2_{\mathrm{L}^2}}{\mu_{V_{\mathrm{i}}^2}} \end{aligned} \tag{4}$$

where $t \in [T - T_{\mathrm{HB}}, T]$, that is by considering the last heartbeat.

Pressures p_{i} and volumes V_{i} are numerical solutions of model $\mathcal{M}_{\text{ANN}}$, while observations $\hat{p}_{\mathrm{i}}$ and $\hat{V}_{\mathrm{i}}$ may come from either in silico numerical simulations or clinical data. In particular, in this work we only focus on observations coming from model $\mathcal{M}_{\text{3D-0D}}$. Coefficients ϵ_{i} to ζ_{i} weigh the pressure and volume traces over time. In this paper, we balance the different terms in Equation (4), i.e. we set ϵ_{i} and ζ_{i} to be either 0 or 1 according to the specific test case. The normalization terms $\mu_{p_{\mathrm{i}}^2}$ and $\mu_{V_{\mathrm{i}}^2}$ are defined by averaging the squared values of pressure and volume traces over time, for $t \in [T - T_{\mathrm{HB}}, T]$.

For the sake of clarity, we recall the mathematical formulation for a system of LNODEs here below:

$$\begin{cases} \dfrac{d\mathbf{z}(t)}{dt} = \mathcal{ANN}\left(\mathbf{z}(t), \cos\left(\frac{2\pi(t - AV_{\mathrm{delay}})}{T_{\mathrm{HB}}}\right), \sin\left(\frac{2\pi(t - AV_{\mathrm{delay}})}{T_{\mathrm{HB}}}\right), \boldsymbol{\theta}; \mathbf{w}\right) & \text{for } t \in (0, T_{\mathrm{HB}}], \\ \mathbf{z}(0) = \mathbf{z}_0. \end{cases} \tag{5}$$

During each iteration of the optimization problem, we compute $\frac{dJ}{d\boldsymbol{\theta}}$ to minimize the loss function $J = J(\boldsymbol{\theta})$. We solve Equation (5) forward in time, i.e. for $t \in (T - T_{\mathrm{HB}}, T]$, using an ODE

numerical solver. Then, we solve an adjoint ODE system backward in time by exploiting reverse-mode differentiation [9]:

$$\frac{d\mathbf{a}(t)}{dt} = -\mathbf{a}(t)^T \frac{\partial \mathcal{ANN}}{\partial \mathbf{z}} \left(\mathbf{z}(t), \cos\left(\tfrac{2\pi(t-AV_{\text{delay}})}{T_{\text{HB}}}\right), \sin\left(\tfrac{2\pi(t-AV_{\text{delay}})}{T_{\text{HB}}}\right), \boldsymbol{\theta}; \mathbf{w} \right), \tag{6}$$

being $\mathbf{a}(t) = dJ/d\mathbf{z}(t)$ the adjoint state.

Finally, the gradient of J with respect to $\boldsymbol{\theta}$ reads [9]:

$$\frac{dJ}{d\boldsymbol{\theta}} = -\int_T^0 \mathbf{a}(t)^T \frac{\partial \mathcal{ANN}}{\partial \boldsymbol{\theta}} \left(\mathbf{z}(t), \cos\left(\tfrac{2\pi(t-AV_{\text{delay}})}{T_{\text{HB}}}\right), \sin\left(\tfrac{2\pi(t-AV_{\text{delay}})}{T_{\text{HB}}}\right), \boldsymbol{\theta}; \mathbf{w} \right) dt \tag{7}$$

All vector-jacobian products in Equations 6 and 7 are evaluated using matrix-free methods, automatic differentiation and automatic vectorization. These overall define an efficient numerical strategy accounting for very small memory requirements [2].

We use the Limited-memory Broyden-Fletcher-Goldfarb-Shanno (L-BFGS) algorithm to solve the optimal control problem (3) [30]. We employ the forward Euler method with a fixed time step $\Delta t = 10^{-3}$ s to solve Equations 5 and 6 at each L-BFGS iteration [11]. We remark that the optimization process is constrained according to the model parameters ranges reported in Table 1.

Once we provide pointwise values of model parameters $\boldsymbol{\theta}_{\text{MAP}}$ via MAP estimation, we evaluate the uncertainty of these estimated values by means of Hamiltonian Monte Carlo (HMC) [8]. This method for inverse UQ allows to find an approximation of either the marginal or joint posterior distribution $\mathbb{P}(\boldsymbol{\theta}|\mathbf{x})$ over $\boldsymbol{\theta}$.

Let $\boldsymbol{\rho} \in \mathbb{R}^{N_\rho}$ be a vector containing auxiliary momentum variables. We define the conditional probability distribution of $\boldsymbol{\rho}$ given $\boldsymbol{\theta}$ as [8]:

$$\mathbb{P}(\boldsymbol{\rho}, \boldsymbol{\theta}) = \mathbb{P}(\boldsymbol{\rho}|\boldsymbol{\theta})\mathbb{P}(\boldsymbol{\theta}),$$

being $\mathbb{P}(\boldsymbol{\theta})$ the prior probability distribution over $\boldsymbol{\theta}$. Then, by employing the kinetic energy $\mathbb{K}(\boldsymbol{\rho}|\boldsymbol{\theta}) = -\log \mathbb{P}(\boldsymbol{\rho}|\boldsymbol{\theta})$ and the potential energy $\mathbb{U}(\boldsymbol{\theta}) = -\log \mathbb{P}(\boldsymbol{\theta})$, we introduce the Hamiltonian function [8]:

$$\mathbb{H}(\boldsymbol{\rho}, \boldsymbol{\theta}) = -\log \mathbb{P}(\boldsymbol{\rho}, \boldsymbol{\theta}) = -\log \mathbb{P}(\boldsymbol{\rho}|\boldsymbol{\theta}) - \log \mathbb{P}(\boldsymbol{\theta}) = \mathbb{K}(\boldsymbol{\rho}|\boldsymbol{\theta}) + \mathbb{U}(\boldsymbol{\theta}).$$

Finally, we solve a coupled system of ODEs in $(\boldsymbol{\theta}, \boldsymbol{\rho})$ to advance the value of the parameters vector $\boldsymbol{\theta} = \boldsymbol{\theta}(t)$ from its current state:

$$\begin{cases} \dfrac{d\boldsymbol{\theta}}{dt} = \dfrac{\partial \mathbb{H}}{\partial \boldsymbol{\rho}} & \text{for } t \in (0, \overline{T}], \\ \dfrac{d\boldsymbol{\rho}}{dt} = -\dfrac{\partial \mathbb{H}}{\partial \boldsymbol{\theta}} & \text{for } t \in (0, \overline{T}], \end{cases} \tag{8}$$

where t represents a fictitious time variable in the parametric space for $\boldsymbol{\theta}$. We solve Equation 8 by means of the leapfrog time scheme and we employ the No-U-Turn Sampler (NUTS) extension of HMC, so that the number of virtual time steps is automatically determined and not user-defined [18]. For the sake of completeness, we report the steps that outline UQ via HMC [8]:

1. we draw a new sample $\boldsymbol{\rho}^0$ from a zero-mean Gaussian distribution for the momentum variables, that is $\boldsymbol{\rho} \sim \mathcal{N}(\mathbf{0}, \boldsymbol{\Sigma})$;

2. starting from $[\boldsymbol{\theta}^0, \boldsymbol{\rho}^0]^T$, we employ the leapfrog numerical scheme and NUTS to solve Equation (8);

3. we negate the momentum variables and define a proposed state $[\boldsymbol{\theta}^*, \boldsymbol{\rho}^*]^T$;

4. we accept the proposed state $[\boldsymbol{\theta}^*, \boldsymbol{\rho}^*]^T$ as the next state using a Metropolis-Hastings update with probability $\min(1, \exp(\mathbb{H}(\boldsymbol{\rho}^0, \boldsymbol{\theta}^0) - \mathbb{H}(\boldsymbol{\rho}^*, \boldsymbol{\theta}^*)))$. On the other hand, if the proposal is not accepted, $\boldsymbol{\theta}^0$ is used again to initialize the next state of HMC.

Once a certain (a priori fixed) number of iterations are made, the accepted proposals for $\boldsymbol{\theta}$, along with the corresponding probability values, are employed to define an approximation of the posterior distribution $\mathbb{P}(\boldsymbol{\theta}|\mathbf{x})$.

We fix $\overline{\Delta t} = 10^{-3}$ and we perform 750 iterations, for all the test cases. Among them, the first 250 iterations consist of an initial burn-in phase and are not retained for the approximation of the posterior distribution $\mathbb{P}(\boldsymbol{\theta}|\mathbf{x})$. We initialize the NUTS sampler by considering $\mathbb{P}(\boldsymbol{\theta}) \sim U(\boldsymbol{\theta}_{\text{MAP}} - \chi\boldsymbol{\theta}_{\text{MAP}}, \boldsymbol{\theta}_{\text{MAP}} + \chi\boldsymbol{\theta}_{\text{MAP}})$, being $\chi = 0.1$ a suitable parameter to define a uniform prior distribution around the MAP estimation $\boldsymbol{\theta}_{\text{MAP}}$. With respect to standard Markov Chain Monte Carlo (MCMC), where multiple chains are usually required to achieve proper convergence to the posterior distribution [44], here we will always run a single chain, as this is sufficient to provide meaningful results. This is also motivated by the suitable initialization of the parameters, which is related to the MAP estimation $\boldsymbol{\theta}_{\text{MAP}}$. We declare convergence when the Gelman-Rubin diagnostic provides a value less than 1.1 for all model parameters and there are no divergent transitions [56].

We account for the surrogate modeling error during the parameter identification process, as all the test cases of this paper are based on time-dependent QoIs coming from model $\mathcal{M}_{\text{3D-0D}}$. We consider normal distributions around the estimated values of these QoIs, i.e. $\mathcal{N}(p_{\text{i}}(t;\boldsymbol{\theta}), \boldsymbol{\Sigma}_{\text{ANN,i}})$ and $\mathcal{N}(V_{\text{i}}(t;\boldsymbol{\theta}), \boldsymbol{\Sigma}_{\text{ANN,i}})$, for i $\in$ {LA, LV, RA, RV}. We introduce a zero-mean Gaussian process $\mathcal{GP}(\mathbf{0}, k(\boldsymbol{t}, \boldsymbol{t}'))$, where $k(\boldsymbol{t}, \boldsymbol{t}') = \sigma^2 \exp\left(\frac{-||\boldsymbol{t}-\boldsymbol{t}'||^2}{2\lambda^2}\right)$ is the exponentiated quadratic kernel [43]. Amplitude σ is independently computed for all the relevant pressure and volume time traces by looking at the pointwise differences of the outputs computed with model $\mathcal{M}_{\text{ANN}}$ and model $\mathcal{M}_{\text{3D-0D}}$ [44]. This leads to $\sigma_{\text{P}_{\text{LA}}} = 0.13$ mmHg, $\sigma_{\text{P}_{\text{LV}}} = 2.30$ mmHg, $\sigma_{\text{P}_{\text{RA}}} = 0.09$ mmHg, $\sigma_{\text{P}_{\text{RV}}} = 0.46$ mmHg, $\sigma_{\text{V}_{\text{LA}}} = 1.82$ mL, $\sigma_{\text{V}_{\text{LV}}} = 1.34$ mL, $\sigma_{\text{V}_{\text{RA}}} = 2.29$ mL and $\sigma_{\text{V}_{\text{RV}}} = 1.38$ mL. The correlation length λ is estimated with 1'000 Adam iterations that minimize the negative log likelihood of the observed surrogate modeling error [21]. We consider a unique value of $\lambda = 0.02$, as we observe similar correlation lengths for all the time-dependent QoIs. The full covariance matrix $\boldsymbol{\Sigma}_{\text{ANN,i}}$ can be then generated by means of the tuned kernel function:

$$\boldsymbol{\Sigma}_{\text{ANN,i}}(t_{\text{j}}, t_{\text{k}}) = \sigma_{\text{i}}^2 \exp\left[\frac{-(t_{\text{j}} - t_{\text{k}})^2}{2\lambda^2}\right] \quad \text{for} \quad \text{i} \in \{\text{LA, LV, RA, RV}\},$$

being t_{j} and t_{k} discrete time points in $[T - T_{\text{HB}}, T]$. We remark that additive measurement errors driven by instrument sensitivities, surrounding environment and human intervention, related to noisy (realistic) observations, may be easily incorporated in our UQ framework as well [47].

In Tables 3-6, we report the true values and median with interquartile range (IQR) for all the estimated parameters in the different test cases ($\mathcal{T}_{\text{LV}}$, $\mathcal{T}_{\text{ventricles}}$, $\mathcal{T}_{\text{atria}}$, $\mathcal{T}_{\text{all}}$), for each numerical simulation of the testing set. We notice that the true parameter value is always properly captured in the range of uncertainty of the corresponding estimation.

In Figure 8, we depict the time evolution of the LV volume for the $\mathcal{T}_{\text{LV}}$ test case, for each numerical simulation of the testing set. The time transients provided by model $\mathcal{M}_{\text{3D-0D}}$ are mostly contained within the 5th and 95th percentiles of the HMC estimations over the 500 samples of the single simulated chain.

In Figure 9, we compare the two-dimensional views of the posterior distribution obtained with test cases $\mathcal{T}_{\text{LV}}$ and $\mathcal{T}_{\text{LV}}^{\text{uniform}}$, i.e. test case $\mathcal{T}_{\text{LV}}$ where HMC is initialized with a uniform prior using

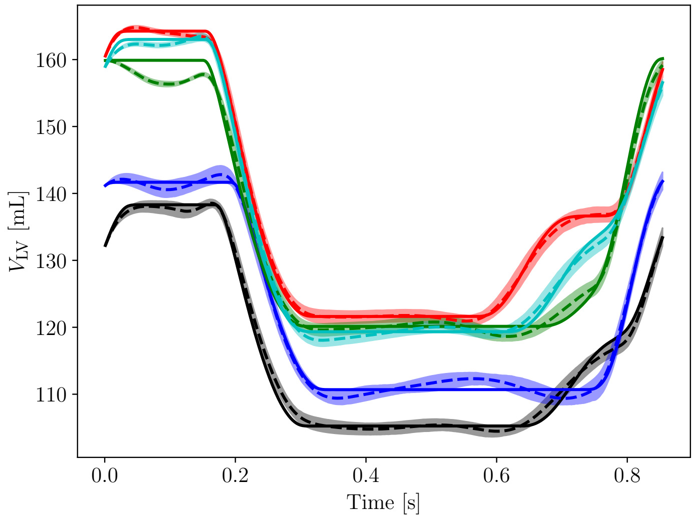

Supplementary Figure 8: $\mathcal{T}_{LV}$: V_{LV} time traces with model $\mathcal{M}_{3D\text{-}0D}$ (solid lines), compared to those obtained with model $\mathcal{M}_{ANN}$ (dashed lines). For the latter, we show the averaged HMC time traces endowed with the 5th and 95th percentiles over the 500 samples of the single chain. Different colors identify different numerical simulations.

the ranges provided in Table 1 instead of a box around MAP. We see that $\mathcal{T}_{LV}^{uniform}$ gives a further estimate of the posterior distribution with respect to $\mathcal{T}_{LV}$, as it potentially requires more than 500 samples on a single simulated chain to reach convergence. In fact, the true parameter values are not always contained within the 95% credibility regions (second row, third column), small isolated blobs appear in the parameter space (second row, first, fourth and fifth columns) and cross-correlations are not always well captured (second row, fifth column). Moreover, even if the HMC initialization is biased toward the MAP estimation, we find that $\mathcal{T}_{LV}$ potentially converges to a multimodal posterior when necessary (first row, third column).

Supplementary Material 7: Speed-up(s) computation

In the main text (Abstract), we state that LNODEs allow for 300x real-time numerical simulations of the cardiac function. This means that simulating one heartbeat, which approximately lasts 1 second, requires 1/300 seconds of execution on a single processor of a standard laptop. Similarly, in the main text (Discussion), we claim that our computational pipeline for global sensitivity analysis and bayesian parameter estimation with UQ based on the surrogate model $\mathcal{M}_{ANN}$ provides a speed-up that is equal to 1718x. This is obtained by dividing the total execution time (508.5 years=185602.5 days) reported in the first part of main text (Table 4), which is related to model $\mathcal{M}_{3D\text{-}0D}$, by the total execution time (108 days) in the second part of main text (Table 4), which is related to model $\mathcal{M}_{ANN}$.

Parameter	Ground truth	Simulation 1	Ground truth	Simulation 2	Ground truth	Simulation 3	Ground truth	Simulation 4	Ground truth	Simulation 5
$dr^{\text{ToRORd-Land}}$	0.23	0.21 (0.05)	0.17	0.17 (0.02)	0.19	0.26 (0.08)	0.13	0.13 (0.02)	0.20	0.21 (0.03)
$ca_{50}^{\text{ToRORd-Land}}$	0.77	0.77 (0.14)	0.53	0.66 (0.15)	0.61	0.79 (0.50)	0.57	0.61 (0.09)	0.51	0.47 (0.11)
R^{sys}	3.28	3.28 (0.49)	2.50	2.26 (0.26)	3.84	3.09 (1.24)	3.57	3.30 (0.34)	2.26	2.77 (0.31)
R^{pulm}	2.63	2.70 (0.43)	2.84	2.64 (0.17)	3.12	2.31 (1.38)	2.45	2.53 (0.18)	3.40	3.02 (0.43)

Supplementary Table 3: $\mathcal{T}_{\text{LV}}$: true value and median with IQR (between brackets) associated to the estimated model parameters for all the $N_{\text{test}} = 5$ electromechanical simulations.

Parameter	Ground truth	Simulation 1	Ground truth	Simulation 2	Ground truth	Simulation 3	Ground truth	Simulation 4	Ground truth	Simulation 5
$dr^{\text{ToRORd-Land}}$	0.23	0.23 (0.03)	0.17	0.16 (0.02)	0.19	0.23 (0.04)	0.13	0.12 (0.02)	0.20	0.21 (0.02)
$ca_{50}^{\text{ToRORd-Land}}$	0.77	0.60 (0.21)	0.53	0.51 (0.17)	0.61	0.62 (0.25)	0.57	0.44 (0.15)	0.51	0.70 (0.21)
$perm_{50}^{\text{ToRORd-Land}}$	0.25	0.32 (0.12)	0.26	0.37 (0.12)	0.37	0.35 (0.15)	0.27	0.35 (0.11)	0.31	0.23 (0.09)
R^{sys}	3.28	3.30 (0.13)	2.50	2.31 (0.20)	3.84	3.73 (0.20)	3.57	3.44 (0.15)	2.26	2.35 (0.19)
R^{pulm}	2.63	2.66 (0.26)	2.84	2.63 (0.32)	3.12	2.81 (0.39)	2.45	2.47 (0.11)	3.40	3.15 (0.28)

Supplementary Table 4: $\mathcal{T}_{\text{ventricles}}$: true value and median with IQR (between brackets) associated to the estimated model parameters for all the $N_{\text{test}} = 5$ electromechanical simulations.

Parameter	Ground truth	Simulation 1	Ground truth	Simulation 2	Ground truth	Simulation 3	Ground truth	Simulation 4	Ground truth	Simulation 5
$dr^{\text{ToRORd-Land}}$	0.23	0.20 (0.05)	0.17	0.15 (0.04)	0.19	0.18 (0.06)	0.13	0.17 (0.05)	0.20	0.20 (0.04)
$perm_{50}^{\text{ToRORd-Land}}$	0.25	0.29 (0.06)	0.26	0.32 (0.07)	0.37	0.45 (0.09)	0.27	0.39 (0.09)	0.31	0.33 (0.06)
$ca_{50}^{\text{CRN-Land}}$	1.09	1.31 (0.15)	1.24	1.19 (0.31)	1.09	0.81 (0.29)	1.06	0.92 (0.28)	0.73	0.66 (0.19)
$TRPN_{\text{n}}^{\text{CRN-Land}}$	2.89	2.17 (0.83)	1.65	2.07 (0.69)	2.14	2.44 (0.90)	1.95	2.18 (0.62)	2.08	2.08 (0.73)
$g_{\text{CaL}}^{\text{CRN}}$	0.13	0.11 (0.03)	0.12	0.13 (0.05)	0.19	0.17 (0.04)	0.14	0.15 (0.04)	0.13	0.17 (0.04)
$b_{\text{t}}^{\text{atria}}$	3.19	3.99 (0.81)	2.40	2.59 (0.61)	2.24	1.71 (0.61)	2.86	2.86 (0.72)	2.50	1.81 (0.84)
R^{sys}	3.28	3.32 (0.27)	2.50	2.30 (0.22)	3.84	3.43 (0.43)	3.57	3.59 (0.32)	2.26	2.68 (0.48)
R^{pulm}	2.63	2.99 (0.49)	2.84	2.62 (0.35)	3.12	2.85 (0.51)	2.45	1.92 (0.62)	3.40	3.19 (0.53)

Supplementary Table 5: $\mathcal{T}_{\text{atria}}$: true value and median with IQR (between brackets) associated to the estimated model parameters for all the $N_{\text{test}} = 5$ electromechanical simulations.

Parameter	Ground truth	Simulation 1	Ground truth	Simulation 2	Ground truth	Simulation 3	Ground truth	Simulation 4	Ground truth	Simulation 5
$dr^{\text{ToRORd-Land}}$	0.23	0.27 (0.04)	0.17	0.15 (0.03)	0.19	0.21 (0.03)	0.13	0.14 (0.02)	0.20	0.22 (0.03)
$perm_{50}^{\text{ToRORd-Land}}$	0.25	0.23 ± 0.12	0.26	0.22 (0.06)	0.37	0.31 (0.15)	0.27	0.31 (0.06)	0.31	0.20 (0.12)
$ca_{50}^{\text{ToRORd-Land}}$	0.77	0.88 (0.15)	0.53	0.63 (0.12)	0.61	0.69 (0.26)	0.57	0.60 (0.13)	0.51	0.69 (0.22)
$ca_{50}^{\text{CRN-Land}}$	1.09	1.02 (0.25)	1.24	1.34 (0.14)	1.09	0.81 (0.31)	1.06	0.79 (0.30)	0.73	0.79 (0.12)
$TRPN_{\text{n}}^{\text{CRN-Land}}$	2.89	3.07 (0.27)	1.65	1.14 (0.56)	2.14	1.79 (0.32)	1.95	1.67 (0.33)	2.08	1.73 (0.36)
$CV^{\text{ventricles}}$	0.44	0.64 (0.20)	0.65	0.61 (0.14)	0.55	0.62 (0.16)	0.50	0.60 (0.13)	0.73	0.84 (0.14)
k_{FEC}	5.88	4.52 (1.39)	3.07	3.53 (1.03)	3.30	2.80 (1.03)	5.61	5.90 (1.06)	2.51	1.46 (1.02)
$g_{\text{CaL}}^{\text{CRN}}$	0.13	0.14 (0.04)	0.12	0.10 (0.03)	0.19	0.14 (0.06)	0.14	0.09 (0.02)	0.13	0.10 (0.04)
$b_{\text{t}}^{\text{atria}}$	3.19	3.32 (0.31)	2.40	2.69 (0.41)	2.24	1.69 (0.43)	2.86	2.35 (0.43)	2.50	3.01 (0.44)
R^{sys}	3.28	3.18 (0.11)	2.50	2.50 (0.06)	3.84	3.76 (0.09)	3.57	3.43 (0.09)	2.26	2.35 (0.10)
R^{pulm}	2.63	2.51 (0.15)	2.84	2.89 (0.08)	3.12	3.07 (0.10)	2.45	2.33 (0.14)	3.40	3.55 (0.16)

Supplementary Table 6: $\mathcal{T}_{\text{all}}$: true value and median with IQR (between brackets) associated to the estimated model parameters for all the $N_{\text{test}} = 5$ electromechanical simulations.

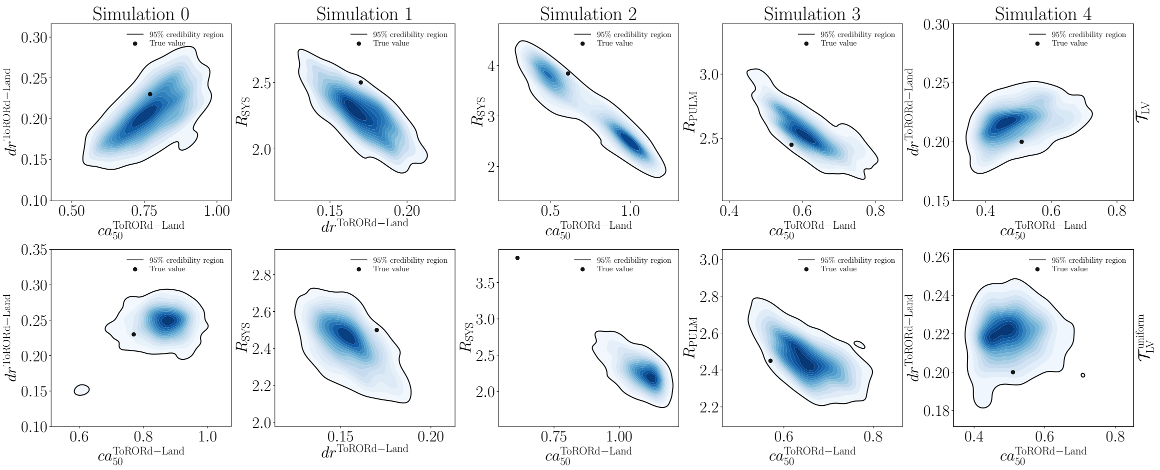

Supplementary Figure 9: Two-dimensional views of the posterior distribution estimated by means of HMC for test cases $\mathcal{T}_{\text{LV}}$ (first row) and $\mathcal{T}_{\text{LV}}^{\text{uniform}}$ (second row) over $N_{\text{test}} = 5$ electromechanical simulations (columns).

		**Pressure**			
		$p_{\text{LA}}(t)$	$p_{\text{LV}}(t)$	$p_{\text{RA}}(t)$	$p_{\text{RV}}(t)$
$\mathcal{M}_{\text{3D-0D}}$ vs $\mathcal{M}_{\text{GPE}}$	NRMSE	0.064	0.028	0.023	0.022
	R^2	95.90	99.70	98.72	99.83
		**Volume**			
		$V_{\text{LA}}(t)$	$V_{\text{LV}}(t)$	$V_{\text{RA}}(t)$	$V_{\text{RV}}(t)$
$\mathcal{M}_{\text{3D-0D}}$ vs $\mathcal{M}_{\text{GPE}}$	NRMSE	0.072	0.217	0.036	0.252
	R^2	97.73	84.44	99.19	63.81

Supplementary Table 7: Testing errors and R^2 coefficients on the time-dependent outputs of the trained GPEs.

Supplementary Material 8: Comparison with Gaussian Processes Emulators

We compare our approach based on LNODEs with Proper Orthogonal Decomposition (POD) [42] combined with Gaussian Processes Emulators (GPEs). For each variable to predict (pressures and volumes over time of LA, LV, RA and RV), we introduce a snapshot matrix $\mathbb{S} \in \mathbb{R}^{N_{\text{train}} \times N_{\text{times}}}$, being $N_{\text{train}} = 400$ the number of numerical simulations in the training dataset and N_{times} the number of time steps for each numerical simulation. The POD of $\mathbb{S}$ is defined as:

$$\mathbb{S} = \mathbb{U}\Sigma\mathbb{V}^T,$$

being $\mathbb{U} \in \mathbb{R}^{N_{\text{train}} \times N_{\text{train}}}$ and $\mathbb{V}^T \in \mathbb{R}^{N_{\text{train}} \times N_{\text{times}}}$ orthogonal matrices and $\Sigma \in \mathbb{R}^{N_{\text{train}} \times N_{\text{train}}}$ diagonal matrix containing the singular values of $\mathbb{S}$. Given the fast decay of the singular values for the pressures and volumes of each cardiac chamber, we define an orthonormal reduced basis $\mathbb{B}$ by truncating

Train/Test	$p_{\text{LA}}(t)$	$p_{\text{LV}}(t)$	$p_{\text{RA}}(t)$	$p_{\text{RV}}(t)$	$V_{\text{LA}}(t)$	$V_{\text{LV}}(t)$	$V_{\text{RA}}(t)$	$V_{\text{RV}}(t)$
400/5	0.028	0.022	0.022	0.021	0.036	0.030	0.054	0.026
395/10	0.030	0.027	0.023	0.025	0.042	0.040	0.055	0.029
385/20	0.031	0.030	0.028	0.030	0.048	0.041	0.055	0.038
365/40	0.034	0.033	0.029	0.031	0.052	0.046	0.057	0.040
400/5	0.028	0.022	0.022	0.021	0.036	0.030	0.054	0.026
395/5	0.036	0.023	0.022	0.023	0.042	0.051	0.057	0.029
385/5	0.037	0.026	0.023	0.024	0.044	0.052	0.060	0.040
365/5	0.040	0.027	0.026	0.028	0.046	0.061	0.073	0.056

Supplementary Table 8: Testing errors on the time-dependent outputs of the trained LNODEs system with different random splittings between the training and testing sets.

matrix $\mathbb{V}^T$ to the first $N_{\text{components}} = 20$ components. In this way, we always explain more than 95% of the variability, which corresponds to a Normalized Root Mean Square Error (NRMSE) below 10^{-5} between the true pressure-volume values and the reconstructed ones via POD reduced basis $\mathbb{B} = \mathbb{V}^T_{\text{truncated}}$. This means that we do not need to train thousands of GPEs on each pressure-volume time point, but we can effectively work in the reduced space. Indeed, we project the snapshot matrix $\mathbb{S}$ onto the reduced basis $\mathbb{B}$. Then, we train multiple GPEs, which provide a statistical model of a scalar map $f(\mathbf{x})$ between $N_{\mathcal{P}} = 43$ physics-based cell-to-organ level parameters and each reduced component. We choose an exponentiated quadratic kernel and we tune each GPE with 1'000 iterations of the Adam optimizer [21]. We use the implementation available in [31]. We evaluate the performance of each GPE on $N_{\text{test}} = 5$ numerical simulations unseen during the training stage. In particular, we compute the NRMSE on the physical space, i.e. by back-projecting via $\mathbb{B}$ the reduced components estimated with GPEs onto the original space. We repeat the training phase of each GPE 5 times by using different initializations and we take the GPE with the best generalization error. We refer to this pipeline as $\mathcal{M}_{\text{GPE}}$. In Table 7, we report the errors and R^2 coefficients for pressures and volumes LA, LV, RA and RV. We notice that the NRMSE is always higher than the corresponding one for LNODEs (see main text, Table 1). In particular, $V_{\text{LV}}(t)$ and $V_{\text{RV}}(t)$ exhibit particularly high values of NRMSE and small values of the R^2 coefficients. This means that more samples are needed especially for these two quantities in order to obtain a reasonable approximation via POD and GPEs. Furthermore, GPEs have to be trained independently on different scalar values while a single system of LNODEs enables time-dependent numerical simulations of cardiac function, capturing cross-correlations among different cardiac chambers. Indeed, in this case, we need to fit 160 scalar GPEs (20 components for 8 physical outputs) to capture the four-chamber heart pressure-volume relationship spanning $N_{\mathcal{P}} = 43$ model parameters. However, tuning a GPE is less computationally expensive than training a system of LNODEs. In fact, we have trained 160 GPEs on a single-core standard laptop in 4 hours, while the optimal system of LNODEs requires 10 hours.

Supplementary Material 9: Increase dimension of the testing dataset

We train the system of LNODEs with different random splittings between the training and testing dataset. We always consider the optimal architecture that we found with hyperparameter tuning,

Train/Test	$p_{\mathrm{LA}}(t)$	$p_{\mathrm{LV}}(t)$	$p_{\mathrm{RA}}(t)$	$p_{\mathrm{RV}}(t)$	$V_{\mathrm{LA}}(t)$	$V_{\mathrm{LV}}(t)$	$V_{\mathrm{RA}}(t)$	$V_{\mathrm{RV}}(t)$
400/5	99.23	99.82	98.85	99.81	99.36	99.50	97.97	99.58
395/10	98.35	99.64	98.42	99.58	98.05	98.69	97.75	99.33
385/20	97.86	99.49	97.38	99.33	97.80	97.95	96.71	98.56
365/40	97.32	99.37	97.13	99.35	96.77	97.15	96.57	98.29
400/5	99.23	99.82	98.85	99.81	99.36	99.50	97.97	99.58
395/5	98.66	99.77	98.85	99.76	99.19	98.92	97.89	99.54
385/5	98.33	99.72	98.69	99.68	98.92	98.84	97.50	99.31
365/5	97.83	99.70	98.45	99.60	98.77	97.98	96.93	99.08

Supplementary Table 9: R^2 coefficients on the time-dependent outputs of the trained LNODEs system with different random splittings between the training and testing sets.

which is equipped with 3 hidden layers and 13 neurons per layer. In Tables 8-9, we report the NRMSE and R^2 coefficients for the different instances. We see that LNODEs retain good generalization properties for all cases. This holds even when the feedforward fully-connected neural network sees only 365 numerical simulation out of 405 numerical simulations during the training phase. Focusing on different training sets and the same 5 test samples, we notice that the NRMSE decreases as the dimension of the training set increases, thanks to the ability of LNODEs to leverage the increasing information content of the dataset. For this reason, in this work we used the LNODEs trained with the largest dataset (i.e. 400) to perform the test cases. We remark that this comes at the price of a reduced size of the testing set, and thus of a less accurate evaluation of the model accuracy on unseen data. Still, Tables 8-9 highlight that, for a fixed number of training samples, the test error evaluated on 5 samples does not significantly depart from the one computed on a larger number of samples (up to 40). The difference is indeed of the order of 0.01, which is within the characteristic level of accuracy considered in this context.

References

[1] M. Abadi, A. Agarwal, P. Barham, and et al. *TensorFlow: Large-Scale Machine Learning on Heterogeneous Systems.* 2015.

[2] P. C. Africa, M. Salvador, P. Gervasio, L. Dede', and A. Quarteroni. "A matrix–free high–order solver for the numerical solution of cardiac electrophysiology". In: *Journal of Computational Physics* 478 (2023), p. 111984.

[3] T. Akiba, S. Sano, T. Yanase, T. Ohta, and M. Koyama. "Optuna: A Next-generation Hyperparameter Optimization Framework". In: *Proceedings of the 25rd ACM SIGKDD International Conference on Knowledge Discovery and Data Mining.* 2019.

[4] T. Arts, T. Delhaas, P. Bovendeerd, et al. "Adaptation to mechanical load determines shape and properties of heart and circulation: the CircAdapt model". In: *American Journal of Physiology-Heart and Circulatory Physiology* 288 (2005), H1943–H1954.

[5] C. M. Augustin, M. A. F. Gsell, E. Karabelas, et al. "A computationally efficient physiologically comprehensive 3D-0D closed-loop model of the heart and circulation". In: *Computer Methods in Applied Mechanics and Engineering* 386 (2021), p. 114092.

[6] J. D. Bayer, R. C. Blake, G. Plank, and N. Trayanova. "A novel rule-based algorithm for assigning myocardial fiber orientation to computational heart models". In: *Annals of Biomedical Engineering* 40 (2012), pp. 2243–2254.

[7] J. Bergstra, R. Bardenet, Y. Bengio, and B. Kégl. "Algorithms for hyper-parameter optimization". In: *Advances in neural information processing systems* 24 (2011).

[8] M. Betancourt and M. Girolami. "A Conceptual Introduction to Hamiltonian Monte Carlo". In: *arXiv:1701.02434* (2017).

[9] R. T. Q. Chen, Y. Rubanova, J. Bettencourt, and D. Duvenaud. "Neural Ordinary Differential Equations". In: *arXiv:1806.07366* (2019).

[10] M. Courtemanche, R. J. Ramirez, and S. Nattel. "Ionic mechanisms underlying human atrial action potential properties: insights from a mathematical model". In: *American Journal of Physiology. Heart and Circulatory Physiology* 275 (1 1998), H301–H321.

[11] J. R. Dormand and P. J. Prince. "A family of embedded Runge-Kutta formulae". In: *Journal of Computational and Applied Mathematics* 6.1 (1980), pp. 19–26.

[12] M. Fedele, R. Piersanti, F. Regazzoni, and et al. "A comprehensive and biophysically detailed computational model of the whole human heart electromechanics". In: *Computer Methods in Applied Mechanics and Engineering* 410 (2023), p. 115983.

[13] P. J. Flory. "Thermodynamic relations for high elastic materials". In: *Transactions of the Faraday Society* 57 (1961), pp. 829–838.

[14] T. Gerach, S. Schuler, J. Fröhlich, et al. "Electro-Mechanical Whole-Heart Digital Twins: A Fully Coupled Multi-Physics Approach". In: *Mathematics* 9.11 (2021).

[15] I. Goodfellow, Y. Bengio, A. Courville, and Y. Bengio. *Deep learning.* Vol. 1. 2. MIT press Cambridge, 2016.

[16] J. M. Guccione and A. D. McCulloch. "Finite element modeling of ventricular mechanics". In: *Theory of Heart.* Springer, 1991, pp. 121–144.

[17] G. A. Holzapfel and R. W. Ogden. "Constitutive modelling of passive myocardium: a structurally based framework for material characterization". In: *Mathematical, Physical and Engineering Sciences* 367 (2009), pp. 3445–3475.

[18] M. D. Homan and A. Gelman. “The No-U-Turn Sampler: Adaptively Setting Path Lengths in Hamiltonian Monte Carlo”. In: *Journal of Machine Learning Research* 15.1 (2014), pp. 1593–1623.

[19] T. Homma and A. Saltelli. “Importance measures in global sensitivity analysis of nonlinear models”. In: *Reliability Engineering & System Safety* 52.1 (1996), pp. 1–17.

[20] E. R. Hyde, J. M. Behar, A. Crozier, and et al. “Improvement of Right Ventricular Hemodynamics with Left Ventricular Endocardial Pacing during Cardiac Resynchronization Therapy”. In: *Pacing and Clinical Electrophysiology* 39.6 (2016), pp. 531–541.

[21] D. P. Kingma and J. Ba. “Adam: A Method for Stochastic Optimization”. In: 2014.

[22] S. Kucherenko, S. Tarantola, and P. Annoni. “Estimation of global sensitivity indices for models with dependent variables”. In: *Computer physics communications* 183.4 (2012), pp. 937–946.

[23] S. Labarthe, J. Bayer, Y. Coudière, and et al. “A bilayer model of human atria: mathematical background, construction, and assessment”. In: *EP Europace* 16 (2014), pp. iv21–iv29.

[24] S. Land and S. A. Niederer. “Influence of atrial contraction dynamics on cardiac function”. In: *International Journal for Numerical Methods in Biomedical Engineering* 34 (2018), e2931.

[25] S. Land, S. J. Park-Holohan, N. P. Smith, et al. “A model of cardiac contraction based on novel measurements of tension development in human cardiomyocytes”. In: *Journal of Molecular and Cellular Cardiology* 106 (2017), pp. 68–83.

[26] A. W. C. Lee, U. C. Nguyen, O. Razeghi, and et al. “A rule-based method for predicting the electrical activation of the heart with cardiac resynchronization therapy from non-invasive clinical data”. In: *Medical Image Analysis* 57 (2019), pp. 197–213.

[27] D. S. Li, E. A. Mendiola, R. Avazmohammadi, and et al. “A multi-scale computational model for the passive mechanical behavior of right ventricular myocardium”. In: *Journal of the Mechanical Behavior of Biomedical Materials* 142 (2023), p. 105788.

[28] L. Li, K. Jamieson, G. DeSalvo, A. Rostamizadeh, and A. Talwalkar. “Hyperband: A Novel Bandit-Based Approach to Hyperparameter Optimization”. In: *Journal of Machine Learning Research* 18.1 (2017), pp. 6765–6816.

[29] L. Li, K. Jamieson, A. Rostamizadeh, and et al. “A System for Massively Parallel Hyperparameter Tuning”. In: *arXiv preprint arXiv:1810.05934* (2020).

[30] D. Liu and J. Nocedal. “On the limited memory BFGS method for large scale optimization”. In: *Mathematical Programming* 45 (1989), pp. 503–528.

[31] S. Longobardi, A. Lewalle, S. Coveney, et al. “Predicting left ventricular contractile function via Gaussian process emulation in aortic-banded rats”. In: *Philosophical Transactions of the Royal Society A: Mathematical, Physical and Engineering Sciences* 378.2173 (2020), p. 20190334.

[32] P. Moritz, R. Nishihara, S. Wang, and et al. “Ray: A Distributed Framework for Emerging AI Applications”. In: *Proceedings of the 13th USENIX Conference on Operating Systems Design and Implementation.* 2018, pp. 561–577.

[33] M. D. Morris. “Factorial sampling plans for preliminary computational experiments”. In: *Technometrics* 33.2 (1991), pp. 161–174.

[34] A. Nasopoulou, A. Shetty, J. Lee, and et al. “Improved identifiability of myocardial material parameters by an energy-based cost function”. In: *Biomechanics and Modeling in Mechanobiology* 16 (2017), pp. 971–988.

[35] A. Neic, F. O. Campos, A. J. Prassl, and et al. “Efficient computation of electrograms and ECGs in human whole heart simulations using a reaction-eikonal model”. In: *Journal of Computational Physics* 346 (2017), pp. 191–211.

[36] A. Neic, M. A. F. Gsell, E. Karabelas, A. J. Prassl, and G. Plank. “Automating image-based mesh generation and manipulation tasks in cardiac modeling workflows using Meshtool”. In: *SoftwareX* 11 (2020), p. 100454.

[37] D. Nordsletten, A. Capilnasiu, W. Zhang, and et al. “A viscoelastic model for human myocardium”. In: *Acta Biomaterialia* 135 (2021), pp. 441–457.

[38] R. W. Ogden. “Nearly isochoric elastic deformations: Application to rubberlike solids”. In: *Journal of the Mechanics and Physics of Solids* 26.1 (1978), pp. 37–57.

[39] D. E. Oken and R. J. Boucek. “Quantitation of Collagen in Human Myocardium”. In: *Circulation Research* 5 (1957), pp. 357–361.

[40] M. Pfaller, J. Hörmann, M. Weigl, et al. “The importance of the pericardium for cardiac biomechanics: from physiology to computational modeling”. In: *Biomechanics and Modeling in Mechanobiology* 18 (2019), pp. 503–529.

[41] E. Plischke, E. Borgonovo, and C. Smith. “Global sensitivity measures from given data”. In: *European Journal of Operational Research* 226.3 (2013), pp. 536–550.

[42] A. Quarteroni, A. Manzoni, and F. Negri. *Reduced Basis Methods for Partial Differential Equations. An Introduction.* Vol. 92. Springer, 2016.

[43] C. E. Rasmussen and C. K. I. Williams. *Gaussian Processes for Machine Learning.* The MIT Press, 2005.

[44] F. Regazzoni, M. Salvador, L. Dede’, and A. Quarteroni. “A machine learning method for real-time numerical simulations of cardiac electromechanics”. In: *Computer Methods in Applied Mechanics and Engineering* 393 (2022), p. 114825.

[45] C. H. Roney, A. Pashaei, M. Meo, and et al. “Universal atrial coordinates applied to visualisation, registration and construction of patient specific meshes”. In: *Medical Image Analysis* 55 (2019), pp. 65–75.

[46] A. Saltelli. “Making best use of model evaluations to compute sensitivity indices”. In: *Computer Physics Communications* 145.2 (2002), pp. 280–297.

[47] M. Salvador, F. Regazzoni, L. Dede’, and A. Quarteroni. “Fast and robust parameter estimation with uncertainty quantification for the cardiac function”. In: *Computer Methods and Programs in Biomedicine* 231 (2023), p. 107402.

[48] I. M. Sobol’. “On sensitivity estimation for nonlinear mathematical models”. In: *Matematicheskoe modelirovanie* 2.1 (1990), pp. 112–118.

[49] E. Song, B. L. Nelson, and J. Staum. “Shapley Effects for Global Sensitivity Analysis: Theory and Computation”. In: *SIAM/ASA Journal on Uncertainty Quantification* 4.1 (2016), pp. 1060–1083.

[50] M. Strocchi, C. M. Augustin, M. A. F. Gsell, and et al. “The Effect of Ventricular Myofibre Orientation on Atrial Dynamics”. In: Springer-Verlag, 2021, pp. 659–670.

[51] M. Strocchi, C. M. Augustin, M. A. F. Gsell, et al. “A publicly available virtual cohort of four-chamber heart meshes for cardiac electro-mechanics simulations”. In: *PLOS ONE* 15 (2020), pp. 1–26.

[52] M. Strocchi, M. A. F. Gsell, C. M. Augustin, et al. "Simulating ventricular systolic motion in a four-chamber heart model with spatially varying robin boundary conditions to model the effect of the pericardium". In: *Journal of Biomechanics* 101 (2020), p. 109645.

[53] M. Strocchi, S. Longobardi, C. M. Augustin, and et al. "Cell to Whole Organ Global Sensitivity Analysis on a Four-chamber Electromechanics Model Using Gaussian Processes Emulators". In: *PLOS Computational Biology* 19 (2023), e1011257.

[54] J. Tomek, A. Bueno-Orovio, E. Passini, et al. "Development, calibration, and validation of a novel human ventricular myocyte model in health, disease, and drug block". In: *eLife* 8 (2019), e48890.

[55] J. Tomek, A. Bueno-Orovio, and B. Rodriguez. "ToR-ORd-dynCl: an update of the ToR-ORd model of human ventricular cardiomyocyte with dynamic intracellular chloride". In: *bioRxiv* (2020).

[56] D. Vats and C. Knudson. "Revisiting the Gelman-Rubin Diagnostic". In: *arXiv:1812.09384* (2018).

[57] J. Walmsley, T. Arts, N. Derval, and et al. "Fast Simulation of Mechanical Heterogeneity in the Electrically Asynchronous Heart Using the MultiPatch Module". In: *PLOS Computational Biology* 11.7 (2015), pp. 1–23.

[58] J. Walpot, D. Juneau, S. Massalha, and et al. "Left Ventricular Mid-Diastolic Wall Thickness: Normal Values for Coronary CT Angiography". In: *Radiology: Cardiothoracic Imaging* 1.5 (2019), e190034.

[59] C. Xu and G. Z. Gertner. "Uncertainty and sensitivity analysis for models with correlated parameters". In: *Reliability Engineering & System Safety* 93.10 (2008), pp. 1563–1573.
